# Eukaryotic cell biology is temporally coordinated to support the energetic demands of protein homeostasis

**DOI:** 10.1038/s41467-020-18330-x

**Published:** 2020-09-17

**Authors:** John S. O’Neill, Nathaniel P. Hoyle, J. Brian Robertson, Rachel S. Edgar, Andrew D. Beale, Sew Y. Peak-Chew, Jason Day, Ana S. H. Costa, Christian Frezza, Helen C. Causton

**Affiliations:** 1 MRC Laboratory of Molecular Biology, Cambridge, CB2 0QH UK; 2 Middle Tennessee State University, Murfreesboro, TN 37132 USA; 3 Molecular Virology, Department of Medicine, Imperial College, London, W2 1NY UK; 4 Department of Earth Sciences, University of Cambridge, Cambridge, CB2 3EQ UK; 5 MRC Cancer Unit, University of Cambridge, Cambridge, CB2 0XZ UK; 6 Columbia University Medical Center, New York, NY 10032 USA; 7 Present Address: Cold Spring Harbor Laboratory, Cold Spring Harbor, NY 11724 USA

**Keywords:** Metabolomics, Proteomics, Saccharomyces cerevisiae, Time series

## Abstract

Yeast physiology is temporally regulated, this becomes apparent under nutrient-limited conditions and results in respiratory oscillations (YROs). YROs share features with circadian rhythms and interact with, but are independent of, the cell division cycle. Here, we show that YROs minimise energy expenditure by restricting protein synthesis until sufficient resources are stored, while maintaining osmotic homeostasis and protein quality control. Although nutrient supply is constant, cells sequester and store metabolic resources via increased transport, autophagy and biomolecular condensation. Replete stores trigger increased H^+^ export which stimulates TORC1 and liberates proteasomes, ribosomes, chaperones and metabolic enzymes from non-membrane bound compartments. This facilitates translational bursting, liquidation of storage carbohydrates, increased ATP turnover, and the export of osmolytes. We propose that dynamic regulation of ion transport and metabolic plasticity are required to maintain osmotic and protein homeostasis during remodelling of eukaryotic proteomes, and that bioenergetic constraints selected for temporal organisation that promotes oscillatory behaviour.

## Abstract (web summary)

Yeast exhibit oscillations that share features with circadian rhythms. The authors show that bioenergetic constraints promote oscillatory behaviour: resources are stored until supplies can support translational bursting, this is licensed by ion transport and release from membrane-less compartments.

## Introduction

*Saccharomyces cerevisiae* undergo oscillations in oxygen consumption and many other cellular processes that synchronise spontaneously when cells are grown at high density in aerobic, nutrient-limited culture at constant pH^1–3^. These metabolic cycles are thought to occur cell-autonomously, and to synchronise when the extracellular nutrient supply is insufficient to support exponential growth^4,5^. Under normal conditions, DNA replication does not occur when oxygen consumption is high, whereas respiratory rate does not affect the timing of mitosis (Supplementary Fig. 1). Thus, yeast respiratory oscillations (YROs) are a population-level phenomenon that is distinct from, and with a different frequency to, the cell division cycle but couples with it by imposing metabolic checkpoints on cell cycle progression^6^. Despite the importance of these oscillations, the mechanism and utility of the YRO is poorly understood^7^.

As with circadian and ultradian (<24 h) rhythms in mammalian cells, YROs are accompanied by large-scale changes in transcription and metabolism, as well as marked changes in the rate of cell growth^8–13^. YROs share many other key features with circadian rhythms in mammalian cells, but have shorter periods that are acutely sensitive to nutrient availability^3,4,7^. For both oscillations, current understanding of the critical causal relationships and rate constants that function over the course of each cycle is inadequate to explain how the period of oscillation is determined^14^.

Here we show that the YRO is defined by phases of higher and lower protein synthesis that drive differential rates of oxygen consumption and are facilitated by profound changes in metabolism and the intracellular ionic environment. These are independent of the cell cycle. Inhibition of translation, changes in pH, potassium concentration, or the abundance of stored carbohydrate, perturb the oscillation. Our data support a model in which cells accumulate metabolic resources and degrade unwanted protein when translation, oxygen consumption and intracellular pH are lower, then liquidate storage carbohydrates and release proteins from membrane-less compartments to support translational bursting and higher rates of respiration, when the pH is high.

## Results

### Cell-intrinsic and -extrinsic factors regulate HOC and LOC

**Fig. 1:**
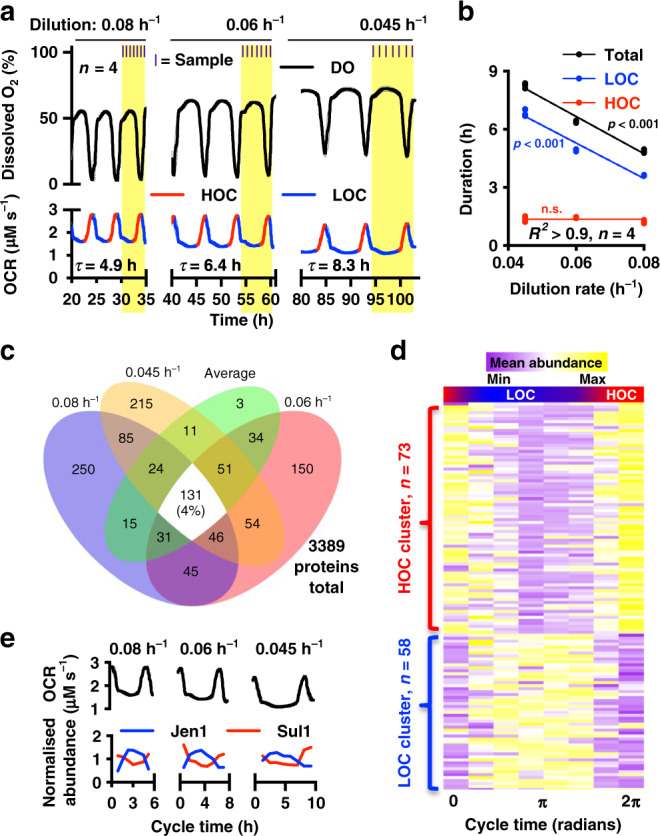
Rhythms in oxygen consumption and protein abundance at three dilution rates. **a** The period (*τ*) of oscillation, which is made up of stages of higher and lower O_2_ consumption (HOC, LOC), varies with dilution rate (mean ± SEM are used throughout, *n* = 4 biologically independent samples). Samples were harvested at the times shown. **b** the duration of LOC, not HOC, varies with dilution rate (extra sum-of-squares *F* test: straight line vs. horizontal line fit, *p*_HOC_ = 0.07, *n* = 4 biologically independent samples). **c** Of 3389 proteins detected by quantitative mass spectrometry, only 4% were consistently rhythmic (varied by >33% across all conditions). **d** Heatmap showing mean abundance of consistently rhythmic proteins clustered with either LOC or HOC (see also Supplementary Data 1). **e** Oxygen consumption and mean-normalized protein abundance, for representative examples of HOC (high-affinity sulfate permease, Sul1) and LOC (monocarboxylate transporter, Jen1) phase proteins. Source data are provided as a Source Data file.

Oxygen consumption rates (OCRs) across the YRO can be interpolated by measuring dissolved oxygen in continuous culture, where phases of higher oxygen consumption (HOC, OCR increases, DNA replication restricted) are distinguished from lower oxygen consumption (LOC) during the rest of the cycle (Fig. 1a, Supplementary Fig. 1). Nutrient availability was manipulated by changing the rate at which medium flowed through the culture (dilution rate); higher dilution rates increase nutrient availability, medium turnover and removal of cells^3^. We noticed that while the period of oscillation and LOC duration lengthened as dilution rate decreased, the duration of HOC was invariant (Fig. 1a, b, Supplementary Fig. 1), suggesting that HOC and LOC are regulated by cell-intrinsic and extrinsic factors, respectively. To explain how YROs are generated, their physiological consequence and identify factors that determine oscillatory period, we sought to understand the differential activities occurring during LOC versus HOC, and at the transition between these two states.

### Rhythmic ion transport, metabolism and cytosolic granules

Previous work suggested that YROs might organise through differentially phased gene expression in order to minimize the cost of expressing large genes^5,8,9,15^, with the expression of up to 60% of cellular mRNAs changing over the course of each cycle^8,9,16^. To gain mechanistic insight, we measured protein, ion and metabolite content by mass spectrometry at multiple time points over the respiratory cycle, from four independent biological replicates at three dilution rates. Given the substantial proteomic coverage, we were surprised to find that only 4% of detected proteins varied with biologically significant amplitudes (>33%^17^) across all three dilution rates. This argues against a central role for dynamic changes in gene expression across the YRO, as does the poor correlation between the rhythmic amplitude, and the stability, abundance, size of each protein or the energetic cost associated with production (Fig. 1c–e and Supplementary Figs. 2 and 3). Of the few proteins whose abundance was rhythmic at the three dilution rates, unbiased k-means analysis suggested two clusters corresponding directly with LOC and HOC (Supplementary Fig. 2a, b), with gene ontology analysis revealing a significant enrichment for terms associated with transmembrane transport of ions and carboxylic acids (Table 1 and Supplementary Fig. 2c, d). For example, the abundance of the sulfate transporter Sul1 clusters with HOC, whereas the monocarboxylate/proton symporter Jen1 clusters with LOC (Fig. 1e and Supplementary Fig. 2d).

**Table 1:** Enrichment for proteins whose abundance changes rhythmically across the YRO reveals the importance of membrane transport.

GO term	Description	*p* value	Enrichment	Activity
0055085	Transmembrane transport	2.31E−06	2.96	**Ena1**, **Pex25**, *Hxt6*, **Vht1**, **Mgr2**, *Jen1*, *Mal11*, *Tim17*, **Fcy2**, *Ato3*, **Thi7**, *Yro2*, **Hip1**, *Ato2*, **Agp1**, **Tim8**, *Itr1*, **Sul1**, *Mdh2*, *Fmp43*, **Ymr166C**, **Yor020W-A**
0015718	Monocarboxylic acid transport	3.71E−05	11.03	*Fmp43*, *Jen1*, **Vht1**, *Ato2*, *Ato3*
0034220	Ion transmembrane transport	4.98E−05	3.54	**Ena1**, *Hxt6*, *Jen1*, *Mal11*, *Ato3*, *Yro2*, **Hip1**, *Ato2*, **Agp1**, **Sul1**, *Fmp43*, **Ymr166C**, **Yor020W-A**

The most highly enriched gene ontology processes for consistently rhythmic proteins, proteins within each GO term are coloured by their YRO phase of expression (bold, HOC; italics, LOC). The complete list of proteins that change in abundance across the YRO is available in Supplementary Data 1. Enriched proteins support the conclusion that membrane transport is important for YROs, likely in response to osmotic stress.

**Fig. 2:**
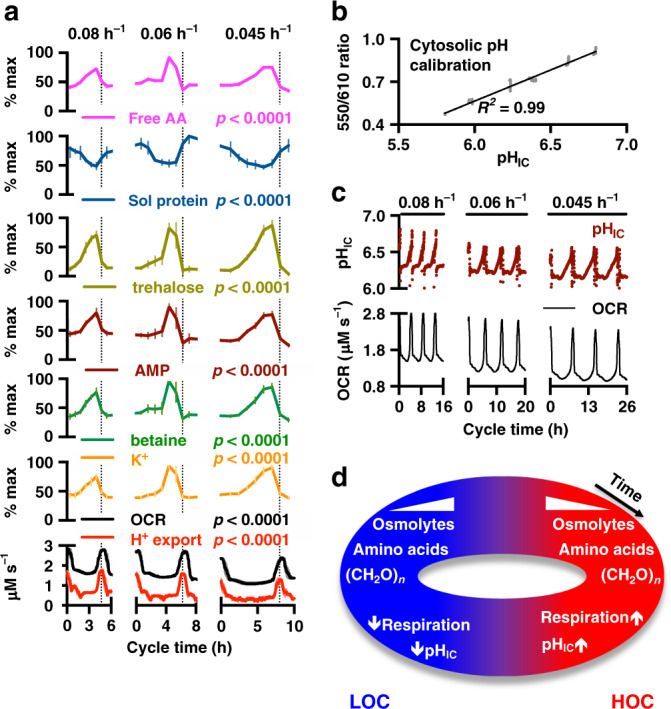
Variation of metabolites, transport, and soluble protein across the YRO. **a** There are consistent phase relationships between intracellular free amino acids (AA), soluble (Sol) protein, trehalose (storage carbohydrate), AMP, betaine, K^+^, OCR and H^+^ export under all conditions (*n* = 4 biologically independent samples, two-way ANOVA_Time_ *p* value shown, mean ± SEM). **b** Calibration curve for firefly luciferase emission ratiometric reporter of intracellular (cytosolic) pH. **c** Intracellular pH (pH_IC_) oscillates as a function of YRO phase under all conditions (representative data). **d** Summary of key events that occur during HOC and LOC. Source data are provided as a Source Data file.

Our proteomic analyses suggested an unexplored requirement for differential ion transport during the YRO. Consistent with this, elemental and metabolite analysis revealed striking >2-fold variations in cellular osmolytes (K^+^, betaine, choline) over the oscillation, which accumulated during LOC and decreased during HOC (Fig. 2a and Supplementary Figs. 4 and 5). Increased osmolyte export was also detectable in the extracellular media, resulting in transient spikes in osmolality during HOC (Supplementary Fig. 5c).

Most active transport in yeast occurs through proton-coupled secondary active transport, driven by a difference of 3 pH units across the plasma membrane, that functions similarly to Na^+^ in mammalian cells^18^. This ~1000-fold gradient is generated by the essential ATP-dependent H^+^-pump, Pma1, which constitutes 15–20% of all yeast membrane protein^19^ and consumes 20–40% of cellular ATP^20^. The large variation in cellular osmolyte content over the YRO strongly suggested differential rates of H^+^ export. To test this, we derived the H^+^-export rate from the volume of NaOH required to maintain constant extracellular pH, as a proxy for Pma1 activity. Our data revealed a robust rise in cellular H^+^-export during HOC, which increased >2-fold in parallel with oxygen consumption, within each cycle (Fig. 2a and Supplementary Fig. 4a, b). Cytosolic pH functions as a second messenger in yeast^21^, so we investigated whether intracellular pH mirrored our observed changes in H^+^-export. To do this we developed a ratiometric luciferase-based assay for real-time measurement of cytosolic pH over the YRO (Fig. 2b). We found that increased H^+^ export was consistently accompanied by a significant rise in intracellular pH (pH_ic_) of >0.5 units (>3-fold drop in [H^+^]), irrespective of dilution rate (Fig. 2c), which rapidly returned to the lower pH at the HOC-to-LOC transition.

In agreement with previous work^22^, >90% of other cellular metabolites we detected showed consistent variation across the YRO at each dilution rate (Supplementary Fig. 5), and unbiased k-means analysis again suggested two clusters, corresponding to HOC and LOC. Metabolites in the major cluster increased during LOC and fell during HOC (Supplementary Fig. 5a, d). Of particular note, amino acids and storage carbohydrates such as trehalose, and indicators of low energy charge such as AMP, all showed similar profiles to cellular osmolytes—increasing by 2–3-fold during LOC and then decreasing in HOC (Fig. 2a), whereas acetate and intermediates of phospholipid synthesis showed opposite profiles (Fig. 2d).

During glucose limitation or osmotic stress, yeast sequester proteins and mRNAs within non-membrane-bound ribonucleoprotein biomolecular condensates (BMCs), including stress granules and p-bodies, whose formation is stimulated by a fall in pH_ic_, and glucose starvation, and maintained by high osmotic potential^23,24^. We predicted that the dramatic decrease in cellular osmolyte content and increase in intracellular pH during HOC would increase the fraction of soluble protein liberated from BMCs. We observed >2-fold variation in soluble cellular protein content, coinciding with HOC, whereas total cellular protein and protein in the media showed no significant variation (Fig. 2a and Supplementary Fig. 6a, b). Gene ontology analysis of soluble proteins with greatest variation between HOC and LOC revealed significant enrichment for protein chaperones, proteasomes, ribosomal subunits and key metabolic enzymes such as enolase (Eno1/2), pyruvate kinase (Cdc19) and decarboxylase (Pdc1) (Supplementary Fig. 6c). These proteins have previously been found in BMCs^24–26^.

### A testable model of the YRO

To explain our observations, we considered that protein synthesis is the most energetically expensive process that cells undertake, requiring high ATP turnover and tRNAs charged with amino acids^27^. The assembly of macromolecular protein complexes (e.g., ribosomes, proteasomes, electron transport complexes) is particularly challenging since subunits must be expressed stoichiometrically, at the same time, and without exceeding chaperone capacity, otherwise they are wastefully degraded or misfold and aggregate, with associated fitness costs^27–29^. Protein synthesis, folding and complex assembly are also sensitive to molecular crowding and osmolyte concentration^24,30^. Indeed, hyperosmotic challenge leads to degradation or sequestration of macromolecular solutes and export of osmolytes to maintain osmotic potential^31,32^. Since macromolecular complexes account for ~40% of all cellular protein^33,34^, efficient complex assembly is particularly challenging for yeast under nutrient-poor conditions, when cells carry out autophagy to generate free amino acids that serve as metabolic substrates for ATP production and accumulate carbohydrate stores of glycogen and trehalose. These stores are rapidly mobilised to fuel anabolic metabolism and cell cycle progression upon the return to growth^35,36^.

Target-of-Rapamycin Complex 1 (TORC1) is the master regulator of protein homoeostasis that controls the switch between anabolic protein synthesis and catabolic autophagy^37–39^. In yeast, TORC1 activity is regulated by glucose via increased pH/Gtr1 signalling, and indirectly by amino acid availability and energy charge via Gcn2 and Snf1/AMPK, respectively^21,26,39,40^. TORC1 activity is also sensitive to molecular crowding, pH and osmolality^30,38,41^. Coincidence detection through TORC1 ensures that the high translation rates required for efficient protein complex biogenesis only occur when sufficient energetic and biosynthetic resources are available.

**Fig. 3:**
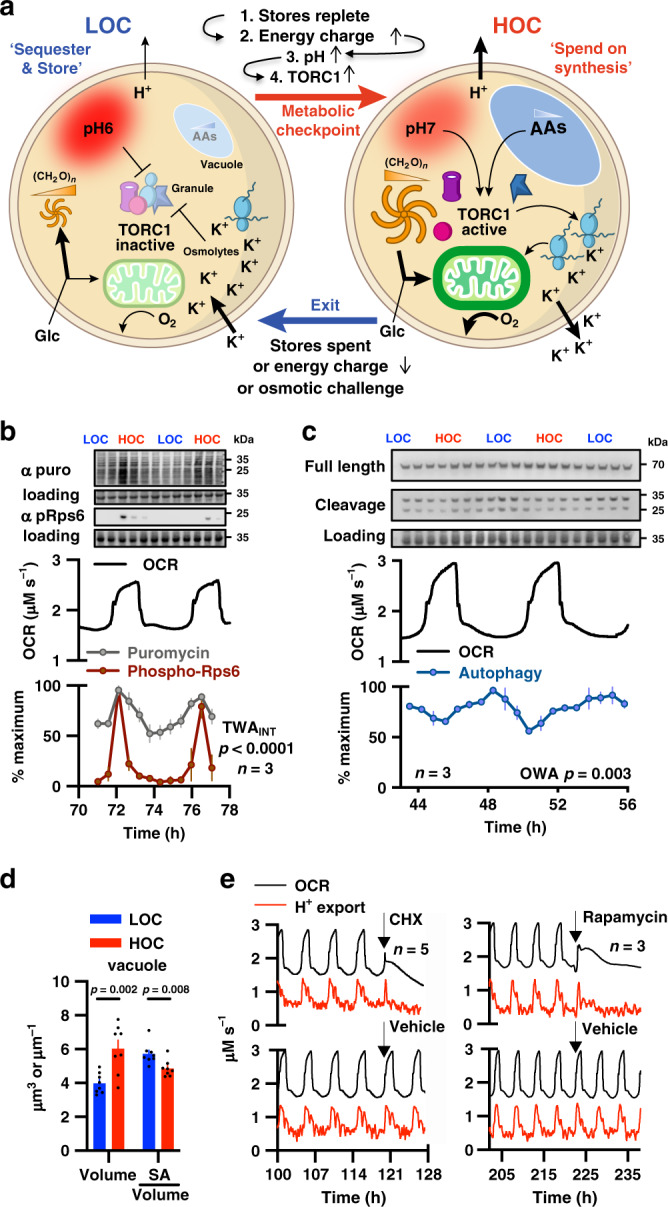
Switching occurs between protein synthesis (HOC) and autophagy (LOC). **a** Model: cells accumulate carbohydrates (CH_2_O)_*n*_, amino acids and osmolytes during LOC and consume/export them in HOC to sustain translational bursts and maintain osmostasis. Replete stores increase H^+^ export, a pH-dependent checkpoint activating TORC1 and releasing BMC proteins. HOC ends when stores are exhausted, see Fig. 5e and Supplementary Table 1 for more details. **b** Puromycin incorporation assay and immunoblot for TORC1 activation (phospho-Rps6, Ser235/236) reveal translational bursting in HOC (*n* = 3 biologically independent samples, TWA_INT_: two-way ANOVA_INTERACTION,_ total protein loading control). **c** Immunoblots for cleaved/full-length Pgk1-GFP reveal increased autophagy during LOC (*n* = 3 biologically independent samples, *OWA* one-way ANOVA, total protein loading control). **d** Differential variation in vacuole volume vs. surface area:volume ratio (two-sided unpaired *t*-test, *n* = 7 independent experiments for HOC and LOC, *n* > 68 cells per image). Data throughout presented as mean ± SEM where *p ≤ 0.05, **p ≤ 0.01, ***p ≤ 0.001, ****p ≤ 0.0001. **e** Acute inhibition of protein synthesis (CHX, 25 µg mL^−1^ cycloheximide or TORC1 activity 200 nM rapamycin) during HOC immediately terminates HOC and abolishes the YRO, representative OCR and H^+^-export traces are shown. Source data are provided as a Source Data file.

In light of these well-established features of yeast cell biology, our observations suggest a mechanistic basis for understanding YROs, where oscillations ultimately arise from the selective pressure for efficient protein synthesis when nutrients are limiting (Fig. 3a). In this model, LOC is the default sequester and store state, in which low amino acid availability, energy charge and pH_ic_ inactivate TORC1, thus facilitating ATP generation through respiration of acetate and autophagic products while protein synthesis is low^21^. Low pH_ic_ also promotes progressive sequestration of macromolecules and glycolytic enzymes, such as Cdc19, into BMCs^26^ and cytosolic removal of these colloidal macromolecular solutes stimulates osmolyte accumulation to maintain osmotic homoeostasis. Respiration of acetate, autophagic and residual glycolytic products generate sufficient ATP to fuel basal Pma1-mediated H^+^ export, but reduced activity of sequestered glycolytic enzymes redirects the bulk of available glucose towards polysaccharide, lipid and nucleotide biosynthesis. The latter two are favoured because more glucose is available for NADPH and ribose production via the pentose phosphate pathway, increasing production of the essential building blocks for cell growth during the G_1/2_ and S-phase of the cell cycle, respectively.

When carbohydrate stores are replete, surplus glucose and ATP stimulates increased H^+^ export by Pma1 (ref. ^42^), thereby raising pH_ic_. Increased pH_ic_ triggers the checkpoint for entry into HOC by stimulating a feed-forward switch that liberates glycolytic enzymes such as Cdc19 from BMCs to increase glycolysis, increasing energy charge and further elevating pH_ic_. Increased pH_ic_ also stimulates TORC1 to license increased protein synthesis, which is sustained because high amino acid levels and ATP:ADP/AMP ratio relieve TORC1 inhibition via the Gcn2 and Snf1/AMPK pathways. Increased translation gradually consumes stored vacuolar amino acids, while energetic requirements are met by liquidation of trehalose and glycogen stores that fuel increased glycolysis and respiration. Liberation of chaperones, proteasomes and other quality control factors from BMCs aids efficient protein synthesis, assembly of protein complexes and turnover of damaged or misfolded proteins. To maintain osmotic homoeostasis, the large increase in osmotic potential that would result from increased cytosolic macromolecular solutes during HOC is buffered by export of osmolytes (K^+^, betaine, choline) to maintain the cytosolic activity of water. In this model, rhythms of respiration and metabolism are ultimately driven by the bioenergetic demands of increased TORC1-stimulated protein synthesis, triggered by elevated pH_ic_. DNA replication and lipid synthesis are restricted during HOC (Supplementary Figs. 1 and 5), because cellular resources are directed towards protein production.

According to our model (Fig. 3a), the end of HOC occurs due to (1) cytosolic acidification due to insufficient ATP (a result of depleted carbohydrate stores and reduced glucose supply), (2) insufficient O_2_ supply which also reduces ATP production and/or (3) exhaustion of amino acid or osmolyte stores, resulting in Gcn2 activation or osmotic challenge, respectively. The period of oscillation is therefore determined by the amount of time taken to replenish osmolyte, amino acid and carbohydrate stores during LOC, and the (normally) invariant duration of HOC reflects a consistent time taken to spend stored carbohydrates, osmolytes and/or amino acids on protein synthesis. We would therefore expect that exit from HOC will be brought forward by acute hyperosmotic stress, translational or respiratory inhibition.

### YRO model validation

This model is consistent with available data and makes many testable predictions. For example, we observed significantly higher rates of protein synthesis and TORC1 signalling during HOC, whereas autophagy was more active during LOC (Fig. 3b, c). Autophagic breakdown products such as free amino acids are stored in the cell vacuole^43^, and we observed significant differences in vacuolar morphology between LOC and HOC corresponding with differential autophagic flux across the YRO (Fig. 3d and Supplementary Fig. 8c).

To test our prediction that the energetic demands of increased protein synthesis drive the characteristic increase in oxygen consumption during HOC, we added cycloheximide (CHX) or rapamycin to cells to inhibit translation or TORC1 activity, respectively. Confirming expectation, both drugs immediately curtailed the anticipated increase in oxygen consumption and proton export during HOC (Fig. 3e), and abolished subsequent oscillations, but without similar effect on basal levels of respiration. At lower, non-saturating concentrations, rapamycin addition during HOC shortened the period of YRO oscillations, but critically the first effect of drug treatment was observed during the next HOC rather than the intervening LOC (Supplementary Fig. 7a, b). By our model LOC is expected to be insensitive to acute TORC inhibition but reduced in length after a truncated HOC, since stores will not be fully depleted and so take less time to replenish. These observations strongly support our hypothesis that TORC1 inactivation suppresses protein synthesis during LOC, and that TORC1 activation triggers the increase in translation that drives the demand for greater respiration during HOC.

**Fig. 4:**
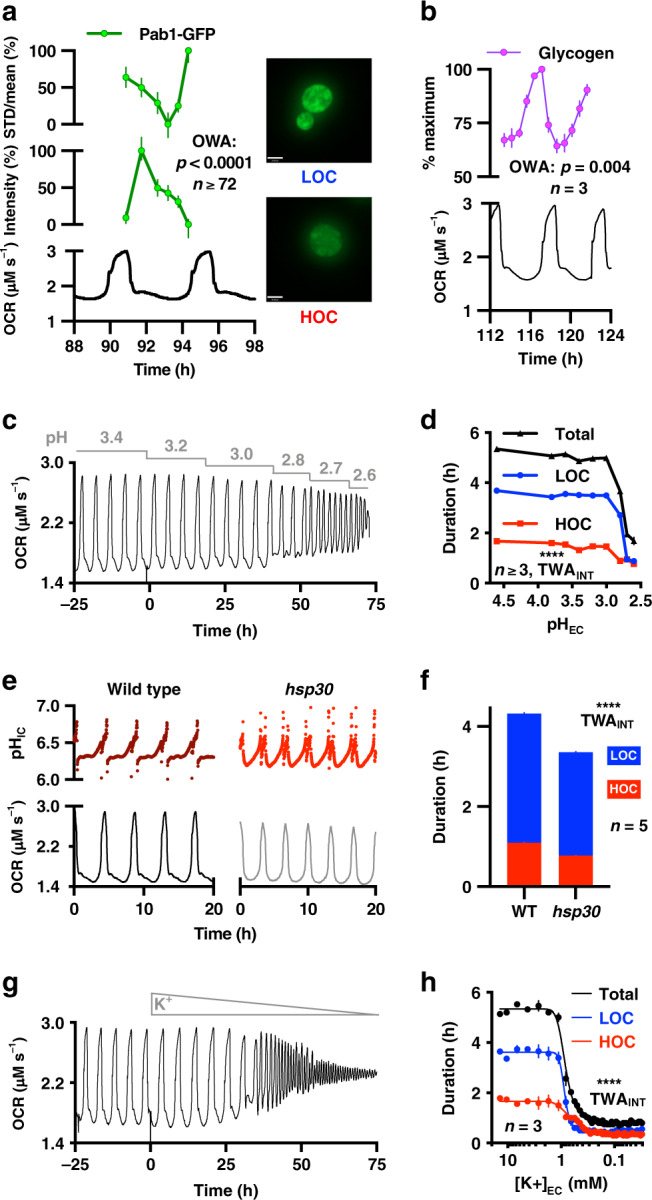
YROs regulate stress granules and glycogen, and are sensitive to H^+^ and K^+^. **a** The intensity and distribution (STD/mean) of stress granule marker Pab1 (Pab1-GFP signal) varies over the YRO, with more foci during LOC and more diffuse during HOC, supporting dynamic variation in stress granule formation (The scale bar represents 1 µm. OWA, n_T90h_ = 4 images and n_T92-97h_ = 8 images one experiment, *n* ≥ 72 granules per time point). **b** Cellular glycogen stores increase during LOC and decrease during HOC (OWA, *n* = 3 biological replicates). Liquidation of storage carbohydrates is likely to fuel translational bursting during HOC. **c**, **d** Decreasing extracellular pH reduces the period of the YRO duration (representative OCR, *n*_pH3.4_ = 4 or *n* = 3 independent experiments). **e**, **f** *HSP30* mutants have truncated oscillations (maroon/red, pH_IC_; black/grey, representative OCR, *n* = 4 biological replicates). **g**, **h** Extracellular K^+^ concentration determines YRO period duration (representative OCR). This is unlikely to be due to loss of cell viability as YROs are rapidly restored when potassium becomes available (representative OCR). All data are shown as mean ± SEM. Source data are provided as a Source Data file.

We next sought to assess the extent of differential BMC sequestration and glycogen storage over the YRO. Consistent with expectation, the stress granule marker, Pab1, was significantly more diffuse during HOC, whereas brighter foci were evident during LOC (Fig. 4a). Moreover, the amount of cellular glycogen fell by 40% during HOC (Fig. 4b), with a profile very similar to that of trehalose (Fig. 2a), the other major storage carbohydrate.

Given the pivotal role of Pma1-mediated H^+^ transport in our model, we tested a prediction that the period of oscillation should be sensitive to increases in pH gradient across the plasma membrane because more ATP turnover will be required to maintain pH_ic_, in both HOC and LOC. Concordantly, we found YROs run >2-fold faster with decreasing, but not increasing, extracellular pH (Fig. 4c, d). Our model also predicts increased Pma1 activity will elicit shorter cycles, as this accelerates the HOC to LOC transition. We tested this with mutants of *hsp30*, a negative regulator of Pma1 (ref. ^44^), observing shorter, truncated cycles of oxygen consumption and pH_ic_ (Fig. 4e, f).

From the model (Fig. 3a), dynamic import and export of osmolytes during LOC and HOC, respectively, is critical for buffering cytosolic osmotic potential: permitting cycles of TORC1 activity and reversible macromolecular sequestration in BMCs/stress granules, both of which are critical for HOC translational bursting and associated respiration increase. Consistent with this, an acute osmotic challenge during HOC, which both inhibits TORC1 activity^30^ and opposes protein liberation from BMCs, resulted in immediate exit from HOC (Supplementary Fig. 7c).

To functionally validate the importance of intracellular osmolyte accumulation to YRO period determination, at the transitions between LOC and HOC, we manipulated the major intracellular osmolyte K^+^. The model predicts that decreased osmotic buffering will shorten the duration of both HOC and LOC. This is because insufficient K^+^ accumulation in LOC means that HOC cannot be sustained, resulting in early osmotic challenge, whereas LOC will be shorter because stored carbohydrates/amino acids were not exhausted during the previous HOC. This, in turn, will reduce the time taken to reach the pH_ic_ threshold for HOC entry in the next cycle. To test this, the infeed was switched to media where K^+^ was replaced with Na^+^. As extracellular K^+^ decreased, we indeed observed a dramatic and reversible shortening of YRO period, whereas depleting Mg^2+^, another essential metal ion but without significant osmotic function, simply abolished oscillations (Fig. 4g, h and Supplementary Fig. 7d, e). Conversely, pulse addition of K^+^ increased YRO period, as well as basal OCR, HOC oxygen consumption and duration, because increased K^+^ availability facilitates greater osmolyte accumulation during LOC allowing HOC to be sustained for longer (Supplementary Fig. 7f, g).

### Physiological consequences

**Fig. 5:**
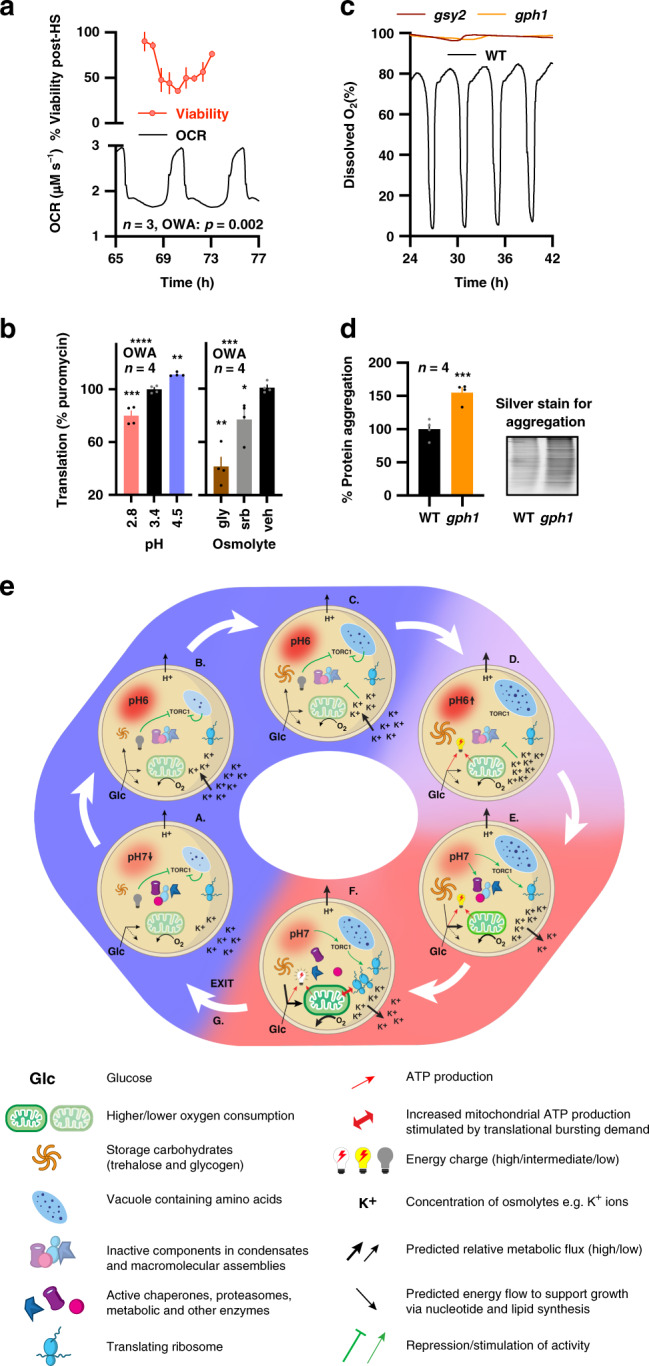
The YRO regulates resistance to heat stress and protein homoeostasis. **a** Viability of cells removed from the bioreactor after heat treatment (55 °C, 2 min) is greatest at the end of LOC, when the abundance of trehalose and osmolytes are greatest. Percentage of heat-treated cells, corrected for viability of non-heat-treated cells harvested at the same time (OWA, *n* = 3 biological replicates). **b** Sensitivity of HOC protein synthesis rate to pH and hyperosmotic stress assayed by puromycin incorporation (gly, 10% glycerol; srb, 1 M sorbitol, *n* = 4 biological replicates). **c** Strains deficient in glycogen synthesis (*gsy2*) or glycogen breakdown (*gph1*) do not initiate YROs and, **d**, *gph1* strains accumulate aggregated protein, showing that glycogen breakdown is necessary for proteostasis. Representative silver-stained gel (two-sided unpaired *t*-test, *n* = 4). **e** A detailed, testable and experimentally derived model for the YRO. Green arrows/lines represent activation/repression, red arrows represent ATP production/stimulation of ATP production, black arrows represent predicted metabolic flux, see key for further details. Data are shown as mean ± SEM. Source data are provided as a Source Data file.

We next explored the physiological consequences of our YRO model, which predicts that the yeast should be more sensitive to a heat stress during HOC, as increased cytosolic protein concentration, reduced osmotic buffering capacity and lower trehalose abundance should render cells more susceptible to protein denaturation. To test this, cells sampled from different points of the YRO were immediately subjected to an acute heat shock, and survival measured under standard growth conditions. Consistent with prediction, a twofold difference in viability was observed between cells harvested at the minimum and maximum OCR (Fig. 5a). We then tested whether translational bursting during HOC is sensitive to acute perturbation of the transmembrane pH gradient and to osmotic challenge, as would be expected. For this assay, we measured protein synthesis by puromycin incorporation. Cells were removed from the bioreactor during HOC and transferred immediately into media of different pH or osmolality. Consistent with expectations, higher pH acutely increased translation whereas lower extracellular pH and hyperosmotic media reduced it (Fig. 5b and Supplementary Fig. 8).

Moving beyond the mechanistic determinants and ramifications of this biological rhythm, our model makes an explicit prediction about its functional utility: translational bursting requires high ATP turnover that can only be sustained by the rapid mobilization of glucose from carbohydrate stores. Therefore, glycogen metabolism mutants will have no temporal organization to the synthesis of proteins or respiration. This will render nascent proteins more likely to exceed chaperone capacity, and/or fail to find a binding partner, and so increase misfolded and aggregated proteins. Consistent with this, glycogen storage or consumption mutants did not exhibit respiratory oscillations and showed a 50% increase in protein aggregation (Fig. 5c, d), indicating a profound deficiency in protein homoeostasis^28,29^.

## Discussion

We provide an experimentally derived, predictive and testable model of the YRO wherein central metabolism, signal transduction, active transport, and macromolecular condensation are temporally organized to accommodate the bioenergetic demands of protein synthesis (Figs. 3a and 5e). This model accounts for the period of oscillation in that HOC duration depends on the amount of time taken to satisfy one or more of the conditions for HOC exit, whereas the duration of LOC depends on the time taken to accumulate carbohydrate.

Eukaryotes have evolved under conditions where nutrients are limiting and environmental conditions vary. Given the high energetic cost of macromolecular complex assembly, and the selective pressure for efficient use of resources, we propose that oscillations in global protein synthesis rate confer a general fitness advantage, realised in many other biological contexts, that is supported by the same save and spend partitioning of metabolic resources we have observed in yeast.

Aspects of our model that involve H^+^ gradients and vacuoles are unlikely to apply to eukaryotic cell types that lack cell walls or use Na^+^ instead of H^+^ gradients. However, we speculate that dynamic transport of cellular osmolytes to buffer osmotic homoeostasis against changes in macromolecular condensation, and dynamic rerouting of metabolic flux to increase ATP production during TORC1-dependent translational bursting, are essential for the temporal organization of proteome remodelling, and therefore common to most eukaryotic cells. Cell-autonomous rhythms of TORC activity, protein synthesis, potassium transport and cellular respiration have been all been observed in mammalian cells over the course of the circadian cycle^45–54^. We therefore speculate that the central utility of biological oscillations such as the YRO, and circadian rhythms throughout the eukaryotic lineage, is to facilitate the efficient utilisation of metabolic resources in order to minimize the cost of protein homoeostasis.

## Methods

### Bioreactor continuous measurement and discrete sampling

Respiratory oscillations were initiated and maintained in a DASgip bioreactor^7,9^. For generation of samples for high-throughput analysis (metabolomics, proteomics, ionomics and flow cytometry) the bioreactor was operated with four replicates in parallel at 220 sL h^−1^ aeration, 550 r.p.m. agitation, 1.5 L media per vessel, at 0.08, 0.06 or 0.045 dilutions h^−1^, 30 °C; pH 3.4 was maintained by addition of 2 M NaOH in media containing 10 g L^−1^ anhydrous glucose, 5 g L^−1^ ammonium sulfate, 0.5 g L^−1^ magnesium sulfate heptahydrate, 1 g L^−1^ yeast extract, 2 g L^−1^ potassium phosphate, 0.5 mL L^−1^ of 70% v/v sulfuric acid, 0.5 mL L^−1^ of antifoam A, 0.5 mL L^−1^ 250 mM calcium chloride, and 0.5 mL L^−1^ mineral solution A (mineral solution A is 40 g L^−1^ FeSO_4_.7H_2_O, 20 g L^−1^ ZnSO_4_.7H_2_O, 10 g L^−1^ CuSO_4_.5H_2_O, 2 g L^−1^ MnCl_2_.4H_2_O and 20 mL L^−1^ 75% sulfuric acid). To initiate YROs bioreactor vessels were each inoculated with 100 OD (*A*_600_) units L^−1^ cells in the stationary phase. The culture was allowed to grow, return to the stationary phase and maintained without addition of new media for 6 h. Oscillations were initiated by refeeding the culture at the dilution rates above and allowed to stabilise for 1–2 days before data collection or harvesting of samples. The dissolved oxygen (DO_2_) traces are shown in Supplementary Fig. 1a. pH was monitored using a calibrated pH electrode and the pH maintained by automated addition of 2 N NaOH. In 1.5 L media, 1 mL h^−1^ 2 N NaOH neutralises H^+^ production equivalent to 0.37 μM s^−1^.

Dissolved O_2_ was monitored with an O_2_ electrode. To establish the relationship between relative DO_2_ and oxygen consumption, the O_2_ electrode was calibrated by allowing anoxic media to equilibrate with atmospheric O_2_ under the conditions described above (100% = 210 μM O_2_ at 30 °C, Supplementary Fig. 1b). This permits OCR (μM s^−1^) to be derived for any steady-state concentration of O_2_ (Supplementary Fig. 1d) where O_2_ is removed from the system. To allow quantitative comparison between experiments, we defined HOC as a sustained increase in OCR such that the 10 min moving average of the first derivative of OCR (dOCR/d*t*) is >0 for ≥10 min and increases by >10% overall within each bout. The phase of LOC occurs between bouts of HOC.

At stated time points, 1.5 mL samples were removed from the bioreactor and harvested by centrifugation (2840*g*, 4 °C, 3 min). The supernatant and pellet were flash frozen separately in liquid nitrogen and stored at −80 °C. Cell pellets (60 µL volume) were then thawed on ice, and washed twice in 1 mL ice cold, isosmotic buffer X (25 mM iodoacetamide, 25 mM NaF, 25 mM NaN_3_, H_2_SO_4_ added to pH 3.4, made up to 150 mOsm with 1 M sorbitol) to remove extracellular ions, proteins and metabolites and inactivate cellular enzymes. Pellets were resuspended in 1 mL buffer X, split into three aliquots, centrifuged, supernatant removed, and stored at −80 °C for analysis by mass spectrometry.

Unless stated, all other bioreactor experiments took place under identical conditions except that 175 sL h^−1^ aeration and 1.0 L media per vessel were used, with appropriate O_2_/pH calibrations and calculations also being performed. Drugs were dissolved in DMSO (Sigma D2650) and added by pulse addition of drug or vehicle to the bioreactor vessel and infeed media. Rapamycin (Sigma R0395) 15 or 200 nM final concentration, CHX 25 μg mL^−1^ (Sigma C7698) was used for the experiements.

### Proteomics

Samples were prepared using 20 µL pellets of yeast, which were lysed in 100 µL lysis buffer (6 M urea, 2 M thiourea, 20 mM HEPES pH 8, with protease and phosphatase inhibitors) with 50 µL of 0.5 mm glass beads by agitation using a Bioruptor Genie (Scientific Industries) at 4 °C (3 × 60 s with 5 min on ice between runs). Lysates were clarified by centrifugation at 21,000*g* for 5 min and total protein concentrations determined using the Pierce BCA assay (Thermo). The concentration of each sample was normalised to 200 µg at 1 mg mL^−1^. For each dilution rate, 100 µL of supernatant from four biological replicates was pooled to generate samples representing nine time points. A pooled cell extract reference sample was made by mixing 25 μL from each of the nine samples.

Samples in 200 μL lysis buffer were reduced with 5 mM DTT at 56 °C, 30 min and alkylated with 10 mM iodoacetamide in the dark at room temperature, 30 min. Samples were then digested with Lys-C (mass spectrometry grade, Promega), 133:1 (protein: Lys-C ratio, w/w) for 4.5 h, 25 °C and then diluted from 8 M to 1.8 M urea with 20 mM HEPES (pH 8.5) and digested with trypsin (Promega) 100:1 (protein: trypsin ratio, w/w) overnight, 25 °C. Digestion was stopped by the addition of trifluoroacetic acid (TFA) to 1% final concentration. Precipitates were removed by centrifugation (9300*g*, 5 min). The supernatants were desalted using home-made C18 stage tips (3M Empore) containing 4 mg poros R3 (Applied Biosystems) resin. Bound peptides were eluted with 30–80% acetonitrile (MeCN) in 0.1% TFA and lyophilized.

Tandem mass tag (TMT) labelling was carried out by resuspending peptide mixtures in 75 μL 3% MeCN and concentrations determined using the Pierce Quantitative Colorimetric Peptide assay (Thermo Scientific) according to the manufacturer’s instructions, except the absorbance was measured using NanoDrop Spectrophotometers (Thermo Scientific) at 480 nm. TMT 10plex reagent (Thermo Fisher Scientific) of 0.8 mg each was re-constituted in 41 μL anhydrous MeCN. In all, 61.5 μL (1.5 × 0.8 mg) of the reagent was used for each sample. The labelling reaction was performed in 150 mM triethylammonium bicarbonate, 1 h at room temperature and terminated by 15 min incubation with 9 μL 5% hydroxylamine. Labelled peptides were combined into a single sample and partially dried to remove acetonitrile in a SpeedVac. The labelled mixture was desalted using C18 stage tips, with 6.6 mg of R3.

Off-line high pH reverse-phase peptides fractionation was carried out using approximately 100 μg of the labelled peptides. These were separated on an off-line, high-pressure liquid chromatography (HPLC). The experiment was carried out using XBridge BEH130 C18, 5 µm, 2.1 × 150 mm (Waters) column with XBridge BEH C18 5 µm Van Guard cartridge, connected to an Ultimate 3000 Nano/Capillary LC System (Dionex). Peptides were separated with a gradient of 1–90% B (A: 5% MeCN/10 mM ammonium bicarbonate, pH 8 [5:95]; B: 90% MeCN/10 mM ammonium bicarbonate, pH 8, [9:1]) in 60 min at a flow rate of 250 µL min^−1^. Sixty fractions were collected, combined into 20 fractions and partially dried in a SpeedVac to about 50 μL.

Liquid chromatography-tandem mass spectrometry (LC-MSMS) was performed on an Ultimate 3000 RSLC nano System (Thermo Scientific) fitted with a 100 µm × 2 cm PepMap100 C18 nano trap column and a 75 μm × 25 cm reverse-phase C18 nano-column (Aclaim PepMap, Thermo Scientific). Samples were separated using a binary gradient consisting of buffer A (2% MeCN, 0.1% formic acid) and buffer B (80% MeCN, 0.1% formic acid). Peptides were eluted with a step gradient of 5–50% B in 87–105 min, 50–90% B in 6–10 min, with a flow rate of 300 nL min^−1^. The HPLC system was coupled to a Q-Exactive Plus mass spectrometer (Thermo Scientific) with a nanospray ion source. The mass spectrometer was operated in standard data dependent mode, performed MS full-scan at 350–1600*m*/*z* range, resolution 140,000. This was followed by MS2 acquisitions of the 15 most intense ions with a resolution of 35,000 and NCE of 32%. MS target values of 3e6 and MS2 target values of 1e5 were used. Isolation window of precursor was set at 1.2 Da and dynamic exclusion of sequenced peptides enabled for 40 s.

The MSMS raw files were processed using Proteome Discoverer (v2.1, Thermo Scientific). MSMS spectra were searched against the reviewed *Saccharomyces cerevisae*, UniProt Fasta database (July 2017), using Mascot (version 2.4, Matrix Science) search engine. Carbamidomethylation of cysteines was set as fixed modification, while methionine oxidation, N-terminal acetylation (protein), phosphorylation (STY) and TMT6plex (peptide N-terminus and lysine) as variable modifications. Other parameters were precursor mass tolerance, 10 ppm and fragment mass tolerance, 0.03 Da. Only peptides with FDR of 1% based on a target decoy approach (high confidence peptides) were included in the results. The output file from Proteome Discoverer, proteins table was filtered for proteins with FDR of 1% and exported as Excel files.

Sample:pooled sample ratio (SPS) was calculated for each time point and normalised so that the median SPS = 1. Sampling times were calculated from dissolved oxygen (DO_2_) measurements and converted into radians where 2*π* radians is equal to the offset time where the correlation coefficient of a serial autocorrelation was at its maximum. Linear interpolation was used on each dataset to calculate the mean SPS across all dilution rates every 0.9 radians. The fold-change between the maximum and minimum SPS detected in the time course for each protein identified in all dilution rates was calculated (FC).

FC versus protein half-life and length in residues was calculated from ref. ^55^. FC versus relative protein abundance and protein cost (relative abundance × length in residues) was also calculated. Protein SPS profiles where FC > 1.33 were clustered (k means, Hartigan Wong algorithm, R version 3.3.3). The between-cluster sum of squares/total within cluster sum of squares was calculated for 1–10 clusters. Due to the lack of inflection points in the plotted data (Supplementary Fig. 2b) we used two clusters for our analysis where the greatest change in cluster/total sum sum-of-squares occurred. GO analysis was performed on each cluster independently, and, when combined, using SGD GO Term Finder version 0.86 involving the process ontology with *p* < 0.05 and default settings.

### Ionomics

Twenty microliters cell pellets were dissolved in 550 µL 65% HNO_3_, supplemented with 100 p.p.b. cerium, at 90 °C for 1 h, then centrifuged at 18,000*g* for 20 min to remove debris. The supernant was diluted 1:12 in HPLC-grade water to give a final matrix concentration of 5% HNO_3_. Cellular elemental composition was determined by inductively-coupled plasma mass spectrometry (ICP-MS) on a PerkinElmer Elan DRC II ICP-MS instrument in helium collision mode. Cerium was used to correct for dilution errors introduced during handling, and then normalized to sodium in buffer X. The operator was blinded to the samples, which were randomised to avoid any effect of machine drift. Ion intensities were calibrated against 10× SPS-SW2 standard (LGC), which was injected nine times during the run. The calibration was checked for accuracy with a second multielement standard purchased from SCP Science (Canada).

### Metabolomics

Cells were collected by centrifugation, the medium discarded and the samples extracted with 200 μL 80 °C hot ethanol. Residual medium resulted in a final ethanol concentration of approximately 80%. The extract was heated for 2 min at 80 °C, vigorously mixed on a vortex mixer and incubated for further 2 min at 80 °C^56^. The extract was cleared of debris by centrifugation and stored at −80 °C for subsequent analysis by liquid chromatography mass spectrometry (LC-MS).

LC-MS analysis was performed on a Dionex U3000 UHPLC system coupled to a Q-Exactive mass spectrometer (Thermo Fisher Scientific). The liquid chromatography system was fitted with a SeQuant ZIC-pHILIC column (150 × 2.1 mm, 5 µm) and guard column (20 × 2.1 mm, 5 µm) from Merck Millipore. The mobile phase was composed of 20 mM ammonium carbonate and 0.1% ammonium hydroxide in water (solvent A), and acetonitrile (solvent B). The flow rate was set at 200 µL min^−1^ with the following gradient: 0 min 80% B, 2 min 80% B, 17 min 20% B, 17.1 min 80% B, and a hold at 80% B for 5 min. Samples were randomized to avoid bias due to machine drift, and the operator was blind to the key. The acquired spectra were analysed using the XCalibur Qual Browser and XCalibur Quan Browser software (Thermo Fisher Scientific) by referencing to an internal library of compounds. Each metabolite is normalised to the total sum of metabolites detected for each sample. ATP was measured enzymatically from cell pellets, extracted as above, by adding 5 µL extract to 95 µL assay mix and compared with an ATP standard curve prepared in parallel. The assay buffer was 30 mM HEPES pH 7.4, 10 mM β-mercaptoethanol, 1 mM potassium luciferin, 10 mM MgSO_4_, and 1 mg mL^−1^ bovine serum albumin and 10 nM QuantiLum (Promega). Luminescence was monitored using a Spark 20 M plate reader (Tecan).

### Soluble protein

Total protein extraction is described above. Soluble proteins were those that dissolved in reducing denaturing buffer following ethanol precipitation, as follows: 20 µL cell pellets were washed twice in 1 mL ice-cold buffer X, then treated with 200 μL 80 °C hot ethanol. The extract was heated for 2 min at 80 °C, vigorously mixed on a vortex mixer and incubated for further 2 min at 80 °C. The extract was clarified by centrifugation, the supernatant removed and the residual pellet washed three times in 1 mL 100% ethanol before being air-dried overnight at room temperature. The dried pellet was resuspended in 200 µL of 8 M urea, 2 M thiourea, 4% CHAPS, 10 mM TCEP and incubated at 37 °C with shaking at 400 r.p.m. for 2 h to dissolve soluble protein, then clarified by centrifugation at 21,000*g* for 10 min. Soluble and total protein concentration was measured by tryptophan fluorescence (excitation: 280 nm, emission: 325 nm) on a Spark 10M plate reader (Tecan), using bovine serum albumin (BSA) to generate a standard curve. Variation in protein content was confirmed qualitatively by SDS-PAGE (Supplementary Fig. 6). Four bands containing soluble protein were excised from denaturing gels, reduced, alkylated and digested with trypsin, using the Janus liquid handling system (PerkinElmer, UK). The digests were subsequently analysed by LC-MS/MS on a Q-Exactive plus orbitrap mass spectrometer (Thermo Scientific, San Jose, USA). LC-MS/MS data were searched against a protein database (UniProt KB) using the Mascot search engine programme^57,58^ (Matrix Science, UK). Proteins of the appropriate molecular weight (>70 kDa for band 1, 40–70 kDa for band 2, 30–40 kDa for band 3 and <30 kDa for band 4) were included for analysis if they were detected at ≥2% abundance for all three dilution rates. GO annotation and enrichment was carried out using the Saccharomyces Genome Database (SGD).

### Osmolality

The osmolality of the media was measured using an OSMOMAT 030 (Gonotec), calibrated with 0 and 300 mOsm standards, according to the manufacturer’s instructions.

### Glycogen content

Cells were harvested by centrifugation, washed with ice-cold phosphate-buffered saline (PBS) and lysed by incubation in 0.25 M sodium carbonate at 98 °C for 4 h. One molar acetic acid and 0.2 M sodium acetate were added to bring the solution to pH 5.2. Glycogen was digested overnight using amyloglucosidase (10115, Sigma-Aldrich) and the glucose released measured using Glucose (GO) Assay Kit (GAGO20, Sigma-Aldrich). Glucose release (mg mL^−1^) was determined from a standard curve and corrected for the OD of starting material.

### Puromycin incorporation

Samples from the bioreactor were immediately incubated with puromycin (AG Scientific, P-1033) at 8 mg mL^−1^ final concentration, 30 min, 30°C in the dark with agitation, before harvesting by centrifugation at 1150*g* for 3 min at 4 °C. For testing the effect of an osmotic challenge, or change in pH, samples were first diluted into an equal volume of media containing 1% glucose plus glycerol or sorbitol at pH 3.4, or into media of different pH, before addition of puromycin, 8 mg mL^−1^ final, and incubation for 20 min at 30 °C.

### Western blot analysis

Yeast whole-cell extracts were prepared as described^59^, except that disruption of the cell wall with glass beads was carried out using a Vortex Genie 2 (Scientific Industries, Bohemia, USA) fitted with a TurboMix head and the protein pellet resuspended in 50 μL Buffer A per OD unit of cells, with protease as described in ref. ^7^. Approximately 10 μg protein was separated by gel electrophoresis in MES buffer on 4–12% bis-tris midi NuPAGE gels (Life Technologies) according to the manufacturer’s instructions. Proteins were transferred to Immobilon-FL (Millipore) in Towbin buffer using a Biorad Transblot Turbo v1.02 blotter set at 25 V, 1.0 A, 30 min, room temperature, stained for total protein using REVERT (LI-COR) before scanning at 700 nm. Blocking was carried out in 1:1 PBS:PBS blocking solution (LI-COR). After incubation with primary and secondary antibodies, the membrane was scanned 800 nm and the results quantified using Image Studio Lite (LI-COR) or IMAGEJ^60^. The signal intensity of total protein/lane was used for normalisation. All graphs were plotted using GraphPad Prism. Western blotting using phospho-specific antibodies was carried out using chemiluminescent detection of horseradish peroxidase (HRP) using TBST containing 0.5% milk and 0.5% BSA.

*Primary antibodies*: mouse monoclonal anti-puromycin (1:5000, Millipore), mouse monoclonal anti-GFP (1:3000, Roche), polyclonal rabbit anti-Phospho-S6 Ribosomal Protein (Ser235/236), recognises Rps6 in yeast and is a readout for TORC1 activity in vivo^61^ (1:1000, Cell Signaling), HRP-conjugated antibodies (1:5000, Sigma), were detected using Luminata Forte (EMD Millipore). IR-labelled secondary antibodies were used at a 1:10,000. Blots were scanned on a gel doc (Bio-Rad) or Odyssey FC (LI-COR).

### Protein aggregation

Aggregated protein was visualised as in ref. ^62^: Wild type and *gph1* cells were grown to exponential phase and ~10 OD units cells (10 *A*_600_ units) harvested by centrifugation. The cell pellet was washed, and resuspended in 300 µL of lysis buffer (50 mM potassium phosphate buffer, pH 7, 1 mM EDTA, 5% (vol/vol) glycerol, 1 mM phenylmethylsulfonyl fluoride, and protease inhibitors: aminobenzamide dihydrochloride (200 µg mL^−1^), antipain(1 µg mL^−1^), aprotinin (1 µg mL^−1^), leupeptin (1 µg mL^−1^), chymostatin (1 µg mL^−1^), PMSF (200 µg mL^−1^), TPCK (50 µg mL^−1^), and pepstatin (1 µg mL^−1^), all from Sigma-Aldrich). The cells were lysed by freezing and thawing followed by incubation (30 °C for 30 min) with 100 µL of lyticase (9830 U mL^−1^) (Sigma-Aldrich). Cells were disrupted with glass beads at 4 °C using a Vortex Genie 2 (Scientific Industries, Bohemia, USA) fitted with a TurboMix head. Intact cells were removed by centrifugation at 3000*g* for 15 min. The membrane and aggregated proteins were isolated by centrifugation at 15,000*g* for 20 min. Membrane proteins were removed by washing twice with 320 µL of lysis buffer and 80 µL 10% Igepal CA-630 (NP-40) (Sigma-Aldrich) and centrifuging at 15,000*g* for 20 min each time. The final aggregated protein extract was resuspended in 100 µL of lysis buffer and analysed by SDS-PAGE. Proteins were visualised by silver staining using the Silver Quest Kit (Invitrogen) and images captured using a Perfection V600 Photo scanner (Epson). ImageJ was used to obtain the background corrected signal intensity per lane^60^.

### Flow cytometry

*Propidium iodide labelling*: 1.5 mL cells (OD 7 to 8) undergoing oscillations in 1.5 L culture were harvested from the bioreactor (4 biological replicates). Aliquots containing ~10^7^ cells were washed in water, resuspended in 500 µL 70% ethanol and stored at 4 °C until use. Samples were harvested by centrifugation at 3381*g*, 4 °C for 10 min. Cell pellets were resuspended in 250 µL Tris buffer pH 7.4 (50 mM) containing RNase A (200 µg mL^−1^) and incubated at 37 °C for 3 h. RNAse-treated cells were harvested by centrifugation at 9391*g* for 5 min and pellets resuspended in 500 µL sodium citrate (50 mM, pH 7.0) before sonication to break up clumps of cells. The volume of sonicated cells was brought up to 500 µL with sodium citrate (50 mM, pH 7.0) containing propidium iodide (Sigma P4170), to give a final concentration of 12.5 µg mL^−1^ and approximately 10^5^ cells mL^−1^. Tubes were put in the dark at 4 °C before processing. Flow cytometry was carried out at the CCTI Flow Cytometry Core on a FACS Canto II (Becton Dickinson). Analysis was performed using the FCS Express density plot function, with the gates shown in Supplementary Fig. 1.

### Ratiometric bioluminescence monitoring of pH_ic_ across the YRO

YROs were established in strains with a stably transformed bioluminescent pH reporter plasmid (either pRS306-PTEF1luc2 or pRS304Nat- PTEF1luc2), 850 mL culture in a 3 L BioFlo 115 benchtop fermentor (New Brunswick) with pH 3.4, 54 L h^−1^ air, 550 r.p.m., and an initial dilution rate of 0.08 h^−1^ (ref. ^63^). Luminescence was continuously recorded using two Hamamatsu PMTs (HC135-01)^64^; however, the PMTs were fitted with 550 ± 5 and 610 ± 5-nm band pass filters (65704 and 65709; Edmund Optics).

After monitoring luminescence from the YRO (as above), continuous culture was terminated and luminescence ratio data generated. The pH of the culture was adjusted to 5.8 by addition of 2 M NaOH. Beetle luciferin (Promega), 5 µM final concentration, and carbonyl cyanide *m*-chlorophenyl hydrazone (CCCP, Promega) was added (1.18 µM final). The culture was incubated for 4 h. The pH was increased stepwise by 0.2 by addition of 2 M NaOH every 15 min covering a pH range 5.8–8.2. Bioluminescence at 550 and 610 nm was continuously monitored as above.

### Confocal microscopy

Samples from the bioreactor were incubated in the dark with 7-amino-4-chloromethylcoumarin (CMAC, LifeTech C2110) 20 µM (final), 30 °C, 10 min with agitation. Cells were washed 1× in media without glucose before imaging. All microscopy was carried out using an Axioskop2 (Zeiss) equipped with a ×100/1.4 Plan-Apochromat objective, Orca ER cooled charge-coupled device (CCD) camera (Hamamatsu) and pE-4000 CoolLED light source at 405 nm, 200 ms for CMAC and 470 nm, 250 ms for GFP (21 image *z* stack at 0.3 μm). Images were deconvolved using iterative restoration and analysed using Volocity 5.3 and 6.5 (PerkinElmer) using default settings.

### Survival after heat stress

Samples from the bioreactor were divided into two aliquots. One hundred and fifty microlitres was incubated at 55 °C for 2 min before serial dilution in media without glucose. The other was diluted in parallel and plated directly (untreated control). The number of colonies/plate was counted after 2 days at 30 °C (median cells per plate 279) and number of colonies on heat-treated vs. non-treated samples expressed as a percentage.

### Osmotic challenge

Potassium chloride (3 M in media without glucose) was added to 200 mM final concentration. Sodium chloride (s), to 1 M final concentration, was added directly to cells in the bioreactor.

### Statistics

Unless otherwise stated, statistical analyses were performed using Prism 8 (Graphpad). Grubbs’ method for outlier detection was employed with *p* = 0.0001 for every species detected by ICP-MS and LC-MS, and found a single consistent outlier (replicate 1, time point 5, dilution rate 0.06 h^−1^) with values that were erroneously high. This was excluded from subsequent analysis.

### Reporter for autophagy

Pgk1/Pgk1-GFP-KanMX strains were made by amplification of the GFP-KanMX cassette from pFA6 plasmid^65^ using primers:

5′-TAAGGAATTGCCAGGTGTTGCTTTCTTATCCGAAAAGAAACGGATCCCCGGGTTAATTAA-3′

5′-CTTAAAATACGCTGAACCCGAACATAGAAATATCGAATGGGAATTCGAGCTCGTTTAAAC-3′ generating an amplicon containing the end of *PGK1*, a 4 amino acid spacer, GFP, KanMX and the 3′ UTR of *PGK1*. This was integrated into the genome of diploid CEN.PK at a single locus. Cleavage of Pgk1-GFP fusion protein by non-selective bulk autophagy can be detected by western blotting^66^.

### Construction of strain with bioluminescent pH_ic_ reporter

pRS306ΔXbaI was created by removing the *Xba*I site in pRS306 (ref. ^67^) by digesting the plasmid with *Xba*I, treating the digested product with DNA polymerase I large fragment and recircularising. The *Bam*HI–*Xho*I fragment from pRS306-PTEF1CBR^64^ containing the *TEF1* promoter, CBR luciferase, and *ADH1* terminator was transferred to the pRS306ΔXbaI plasmid to produce pRS306-PTEF1CBRΔXba. The *luc2* CDS was PCR amplified from pGL4.20 using primers: 5′-actactAGATCTATGGAAGATGCCAAAAACATTA-3′, 5′-actacgTCTAGATTATTTTTCGAACTGCGGGT-3′ adding a *Bgl*II and *Xba*I site upstream and downstream of the start and stop codons respectively. Finally, the *luc2* PCR product was added to pRS306-PTEF1CBRΔXba with *Bgl*II and *Xba*I (replacing the CBR luciferase CDS) and generating pRS306-PTEF1luc2, an integrating plasmid for constitutive *luc2* expression. pRS306-PTEF1luc2 was linearized with *Eco*RV and transformed into yBR-ura3ΔCEN.PK113-7D^68^, creating strain yBR-PTEF1luc2.

A nourseothricin selectable bioluminescent pH reporter was made by putting the *Bam*HI–*Sal*I fragment from pRS306-PTEF1luc2 (containing the *TEF1* promoter, luc2 luciferase, and *ADH1* terminator) into pRS304-Nat^64^ creating pRS304Nat- PTEF1luc2. This plasmid was linearized with *Eco*RV and integrated into the native *TRP1* gene of strain HYC1602.

### Yeast strains

All strains are isogenic *Mata* haploids in the CEN.PK113-7D background unless stated.

HCY1514 *prototrophic wild type*

HCY1602 *HSP30::KanMX*

HCY1648 *GPH1::KanMX*

HCY1674 *GSY2::KanMX*

HCY1325 *Mata/Matα*

HCY1689 to HCY1691 *Mata/Matα PGK1/PGK1*-GFP-KanMX

HCY1800 *Mata* yBR-PTEF1luc2

HCY1803 *Mata* BR-PTEF1luc2 *HSP30::KanMX*

HCY1820 *Mata ura3Δ0* [pRP1657 Pab1-GFP Edc3-mCherry]^69^

Gene inactivation was carried out by amplification of the KanMX antibiotic resistance cassette from either the pFA6 plasmid^65^, or a deletion strain from the Research Genetics Deletion Collection using their A and D primers and integrated into the genome of a CEN.PK strain. The location of the cassette was confirmed by PCR and/or sequencing.

### Reporting summary

Further information on research design is available in the Nature Research Reporting Summary linked to this article.

## Supplementary information

Supplementary Information

Peer Review File

Supplementary Data 1

Reporting Summary

## Data Availability

Proteomic data that support the findings of this study have been deposited in the ProteomeXchange Consortium via the PRIDE partner repository with the identifier PXD013653. Other data that support the findings of this study are available from the corresponding authors upon reasonable request. Source data are provided with this paper.

## Source data

Source Data
