# Author Correction: Eukaryotic cell biology is temporally coordinated to support the energetic demands of protein homeostasis

**DOI:** 10.1038/s41467-021-27497-w

**Published:** 2021-12-08

**Authors:** John S. O’Neill, Nathaniel P. Hoyle, J. Brian Robertson, Rachel S. Edgar, Andrew D. Beale, Sew Y. Peak-Chew, Jason Day, Ana S. H. Costa, Christian Frezza, Helen C. Causton

**Affiliations:** 1 MRC Laboratory of Molecular Biology, Cambridge, CB2 0QH UK; 2 Middle Tennessee State University, Murfreesboro, TN 37132 USA; 3 Molecular Virology, Department of Medicine, Imperial College, London, W2 1NY UK; 4 Department of Earth Sciences, University of Cambridge, Cambridge, CB2 3EQ UK; 5 MRC Cancer Unit, University of Cambridge, Cambridge, CB2 0XZ UK; 6 Columbia University Medical Center, New York, NY 10032 USA; 7 Present Address: Cold Spring Harbor Laboratory, Cold Spring Harbor, NY 11724 USA

**Keywords:** Metabolomics, Proteomics, Saccharomyces cerevisiae, Time series

Correction to: *Nature Communications* 10.1038/s41467-020-18330-x, published online 17 September 2020.

In this article the author name John S. O’Neill was incorrectly written as John S. O’ Neill. This has now been corrected in the HTML and PDF version of this article.

