# Supplementary material for: Eukaryotic cell biology is temporally coordinated to support the energetic demands of protein homeostasis
Source: Nat Commun. 2020 Sep 17;11:4706. doi: 10.1038/s41467-020-18330-x (PMC7499178; doi:10.1038/s41467-020-18330-x)
Supplement: Supplementary file 1 — Supplementary Information [file 41467_2020_18330_MOESM1_ESM.pdf]

Supplementary Information

Eukaryotic cell biology is temporally coordinated to support the energetic demands of protein homeostasis

O'Neill *et al*.

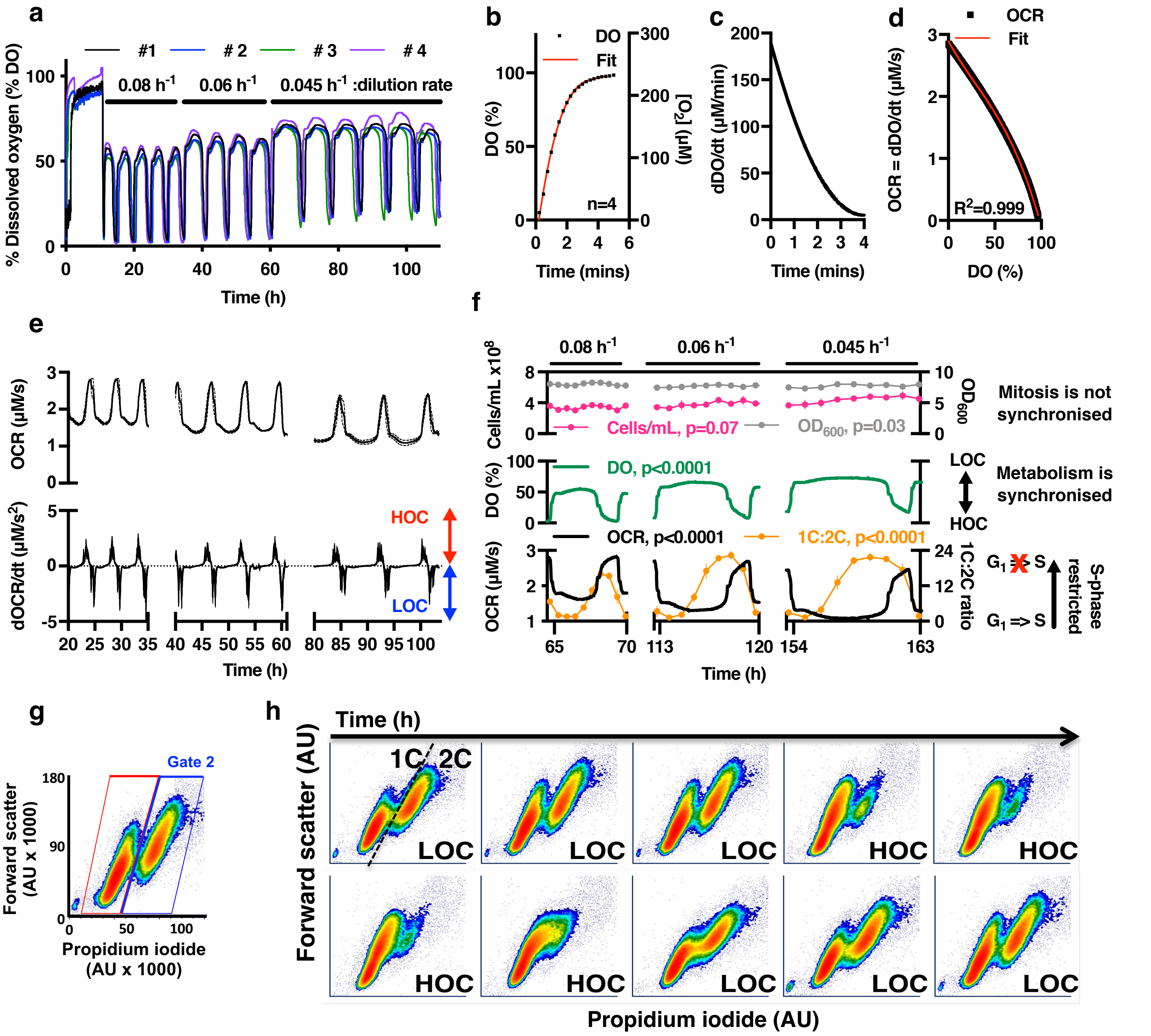

Supplementary Figure 1, Relating to Figure 1. The YRO gates S-phase entry but not mitosis

a, Individual dissolved oxygen traces from the four bioreactor vessels used for harvesting of cells for multi-omic analysis. **b**, Reoxygenation of media (without cells) over time under conditions used for YROs, n=4 independent experiments. **c,** Dissolved O_2 quickly equilibrates with that in the gaseous phase, so under constant aeration the rate at which dissolved O_2 increases, decreases as $[O_2]$ approaches saturation (n=4 independent experiments). **d**, As O_2 is supplied at a constant rate, the rate at which O_2 is consumed (O_2 consumption rate, OCR) to achieve any given steady state $[O_2]$ can be interpolated from the standard curve. **e**, To allow comparison between experiments, we define HOC as the time when the rate of change in oxygen consumption rate is above zero, LOC when below zero. HOC and LOC durations may readily be quantified from the first derivative of OCR (mean±SEM, for the 4 biological replicates shown in a). **f**, DNA replication is inhibited at the transition into and during HOC, resulting in an oscillation in the relative proportion of 1C to 2C cells (G1:G2). There is no significant variation in cell number across the YRO that is consistent for all three dilution rates, however. This means that any variation observed across the YRO at all three dilution rates cannot be attributed simply to synchronized mitosis or variation in cell number (mean±SEM, n=4 biological replicates, 2-way ANOVA for time effect reported). **g**, Gating strategy to determine the percentage of 1C (gate 1) and 2C (gate 2) cells showing the intensity of the propidium iodide signal (DNA) vs. forward scatter for populations. **h**, Representative flow cytometry data of populations of yeast oscillating at 0.08 dilutions/h. Scale is the same as g.

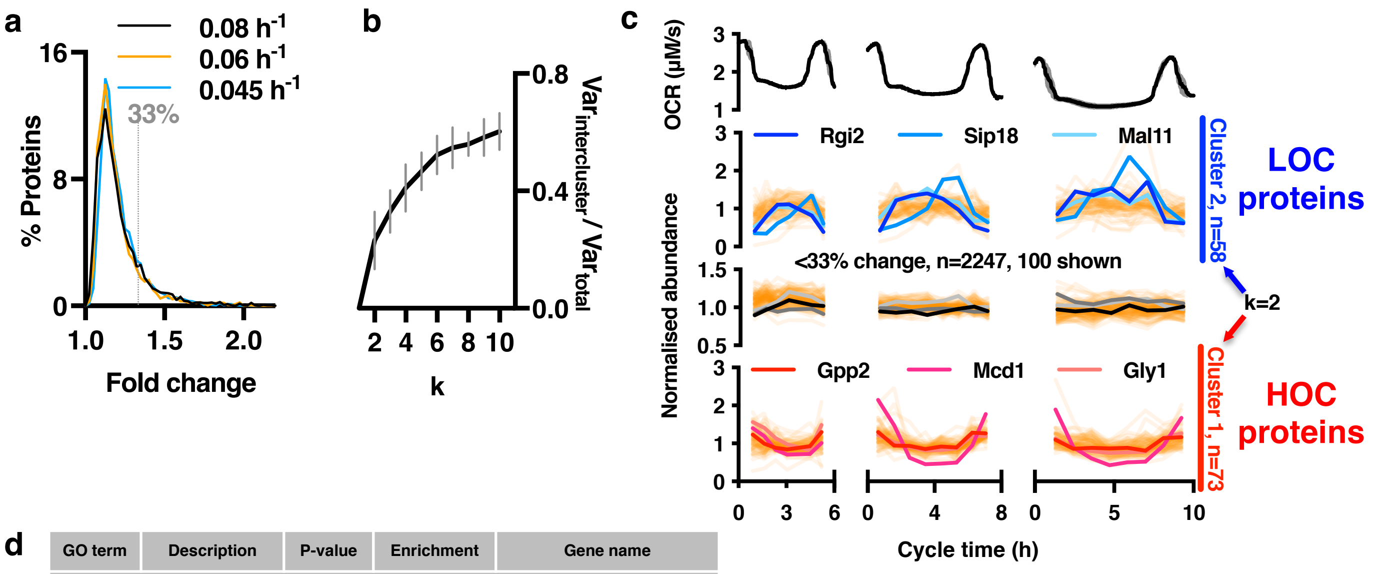

d

GO term	Description	P-value	Enrichment	Gene name
HOC enriched process (>33% amplitude)				
0072348	sulfur compound transport	0.00015	23.76	*SUL1 VHT1 THI7*
0055085	transmembrane transport	0.00044	3.03	*SUL1, VHT1, THI7, ENA1 HIP1, AGP1 FCY2, MGR2, TIM8, YMR166C, YOR020W-A, PEX25*
0006114	glycerol biosynthetic process	0.00063	39.61	*RHR2, HOR2*
LOC enriched process (>33% amplitude)				
0015718	monocarboxylic acid transport	0.00002	21.66	*FMP43, JEN1 ATO2, ATO3*
0010035	response to inorganic substance	0.00010	10.15	*NCE103 CUP1-2, CRS5 SIP18, STF2*

Supplementary Figure 2, Relating to Figure 1. Proteomics reveals modest changes in protein abundance with clusters that correspond to HOC and LOC. a, Plot of fold change vs. detected proteins for three dilution rates. Informed by quantitative genome-wide measurements of the intrinsic noise of gene expression in yeast[1], and given that our approach will not detect very low copy number proteins, we chose a conservative threshold for biological significance of 1.33. This means that any protein whose abundance does not change by >33% over the YRO at all three dilution rates, as well as the average abundance across all dilutions, is not considered to be consistently rhythmic. **b**, Amongst the consistently rhythmic proteins, unbiased k means cluster analysis provided no strong support for any specific number of clusters between 2-10, as revealed by the 'break point' in a plot of inter-cluster variation versus cluster number. We therefore selected the simplest model, i.e., two clusters. The temporal profiles of proteins in these two clusters correspond with the relative phases of HOC and LOC. **c**, Plot of normalized protein abundances at each dilution rate vs. OCR (top, repeated from Figure 1a), stratified by profile. Three randomly selected examples highlight proteins whose expression peaks during LOC or HOC. Note that the majority of detected proteins 2247/3389 varied by <33% at all 3 dilution rates, whereas proteins that were consistently rhythmic typically showed modest changes in abundance. **d**, The most enriched non-redundant Gene Ontology processes for consistently rhythmic proteins in the HOC and LOC clusters reveals differential regulation of transporters in both YRO phases. Also see Supplementary Table 1.

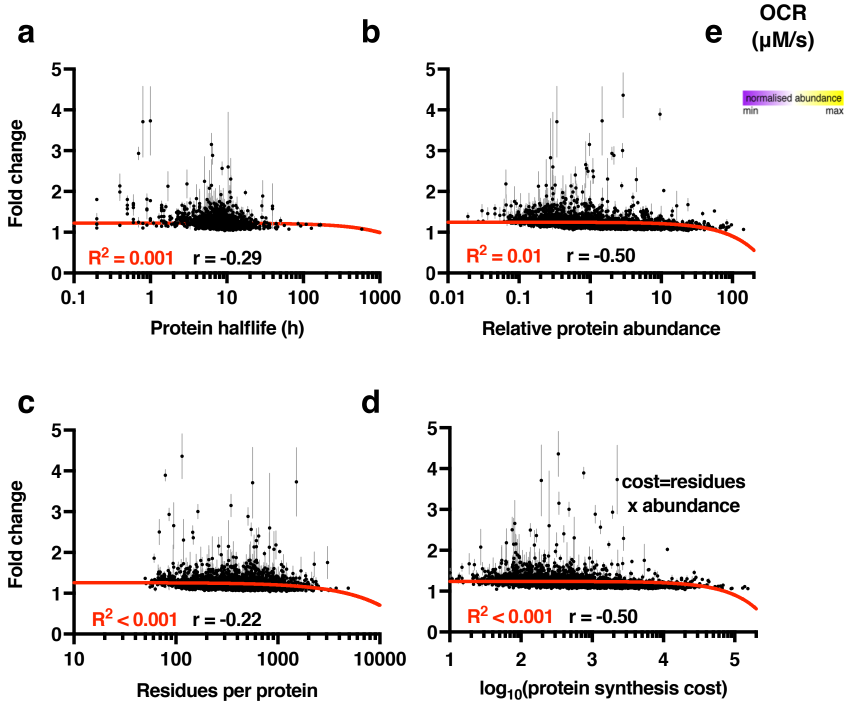

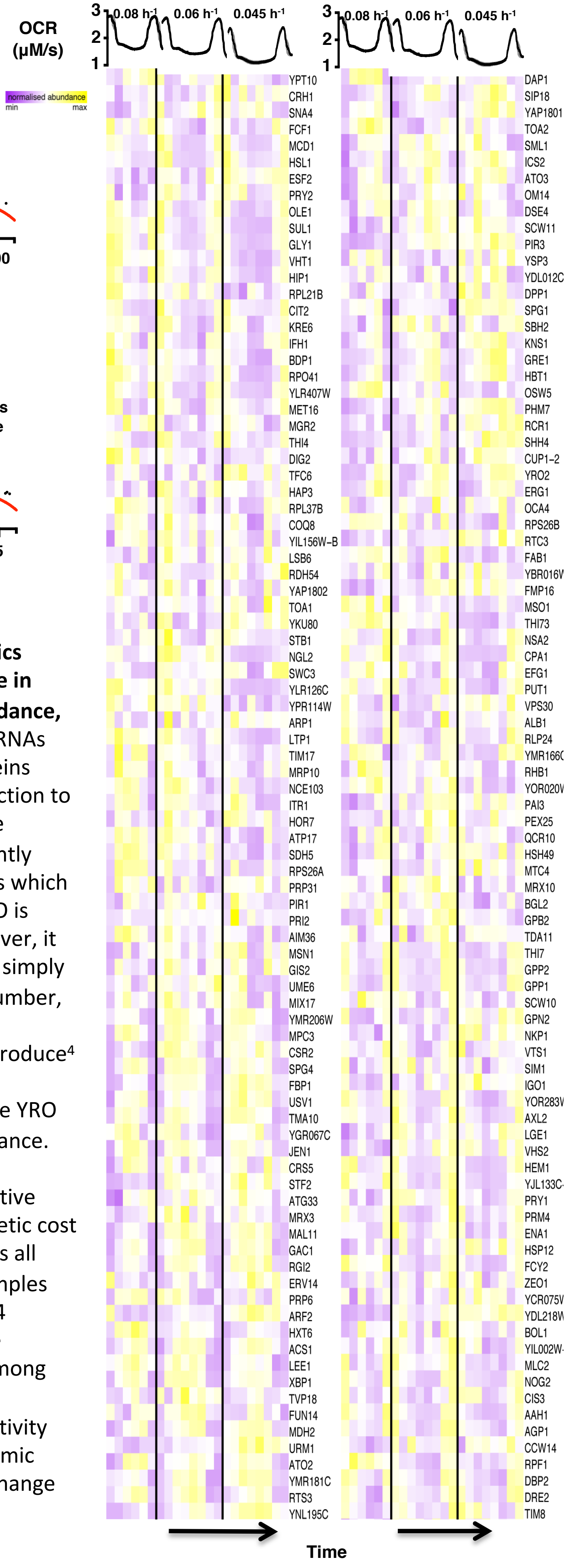

Supplementary Figure 3, Relating to Figure 1. Proteomics analysis reveals weak correlations between fold change in protein abundance across the YRO with stability, abundance, size or cost of synthesis. a-d, The expression of most mRNAs varies over the YRO [2,3], yet the abundance of most proteins does not, implying that transcriptional rhythms may function to maintain steady state protein levels, rather than to drive rhythms in the activity of the encoded protein. Consistently rhythmic proteins were enriched for transport processes which implies that differential transporter activity over the YRO is relevant to its mechanism and biological function. However, it was equally plausible that more rhythmic proteins were simply those with shorter half-life, or those with higher copy number, such that changes in abundance would be more readily detectable. Because larger proteins are more costly to produce[4] another possibility is that natural selection has acted to suppress the production of large proteins at stages of the YRO when they are not needed, leading to rhythms in abundance. Considering all 3389 detected proteins, however, poor correlations were observed between each protein's relative amplitude and its half-life, abundance, size or the energetic cost of its synthesis (size x abundance). Data combined across all three dilution rates (mean±SEM, n=3 pooled protein samples from 4 biological replicates across 3 dilutions). Since all 4 simpler hypotheses were rejected by these analyses, we consider that the over-representation of transporters among consistently rhythmic proteins occurs in response to the requirement for differential regulation of transporter activity over the YRO. **e**, Heatmap showing all consistently rhythmic proteins without clustering, normalized to the relative change for each protein over the course of the oscillation.

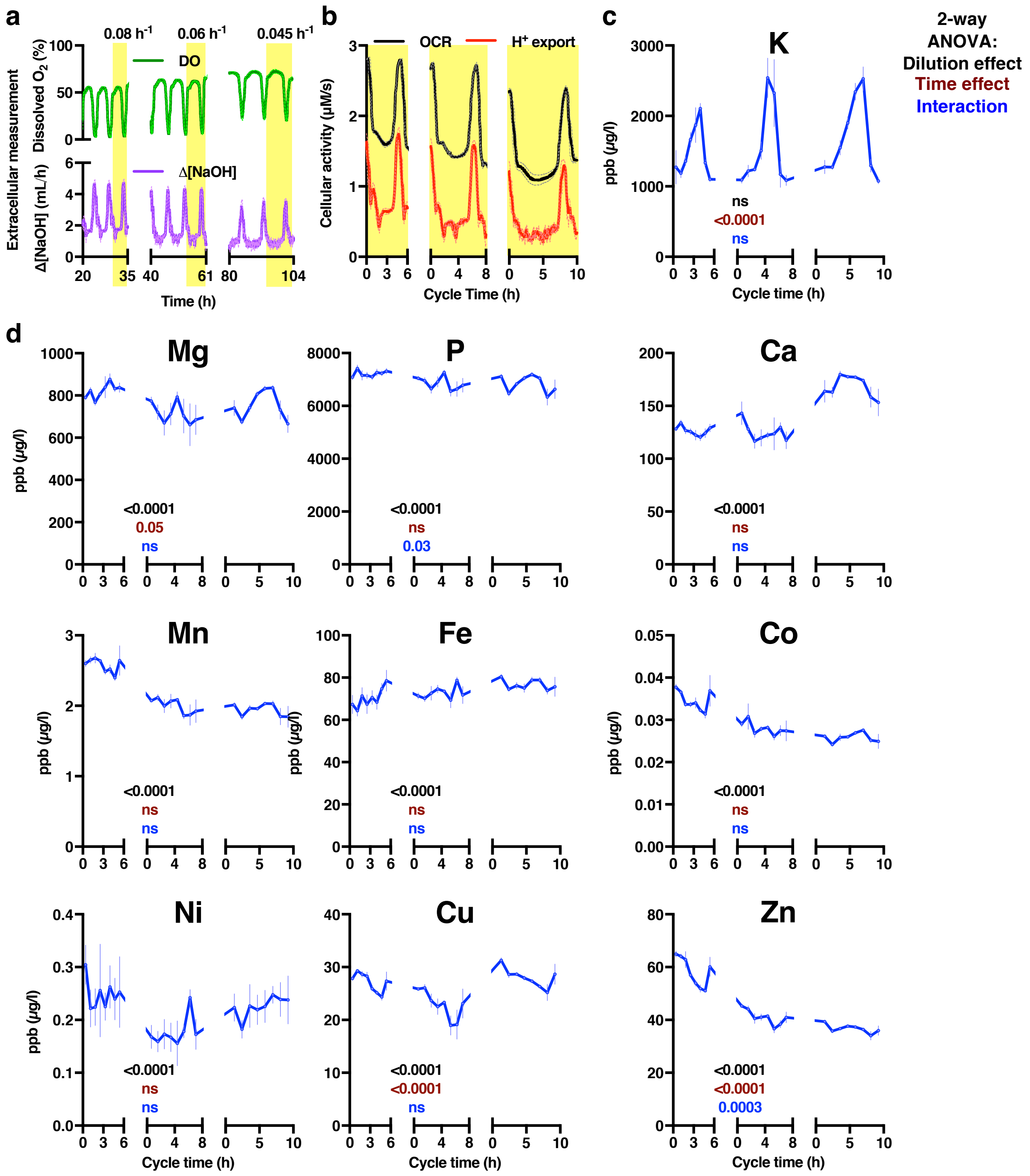

Supplementary Figure 4, Relating to Figure 2. Cellular ionomics, H^+ and K^+ export vary consistently over the YRO **a**, NaOH is pumped into bioreactor vessels to maintain the media at pH 3.4; greater NaOH addition occurs during HOC compared with LOC (mean±SEM, n=4 biological replicates). **b**, From the rate of NaOH addition and its concentration, the rate at which the cell population exports H^+ is readily interpolated. H^+ export rate changes in parallel with the oxygen consumption rate (OCR) (mean±SEM, n=4 biological replicates). **c**, and **d**, Samples of cells undergoing YROs were harvested across four replicates at three dilution rates, as described, and subjected to analysis of intracellular metal ion content by ICP-MS using ^{43}Ca, ^{44}Ca, ^{59}Co, ^{63}Cu, ^{65}Cu, ^{57}Fe, ^{39}K, ^{24}Mg, ^{55}Mn, ^{60}Ni, ^{31}P, and ^{66}Zn. Only the intracellular concentration of potassium ions shows variation significant for time but not dilution or interaction effect (mean±SEM, n=4 biological replicates, 2-way ANOVA reported). N.B. Due to the preparative method used for these yeast cell samples, Na^+ and Cl^- could not be measured (this method was used to make them compatible with the metabolomic and proteomic data).

Supplementary Figure 5(i)

Cellular metabolomics

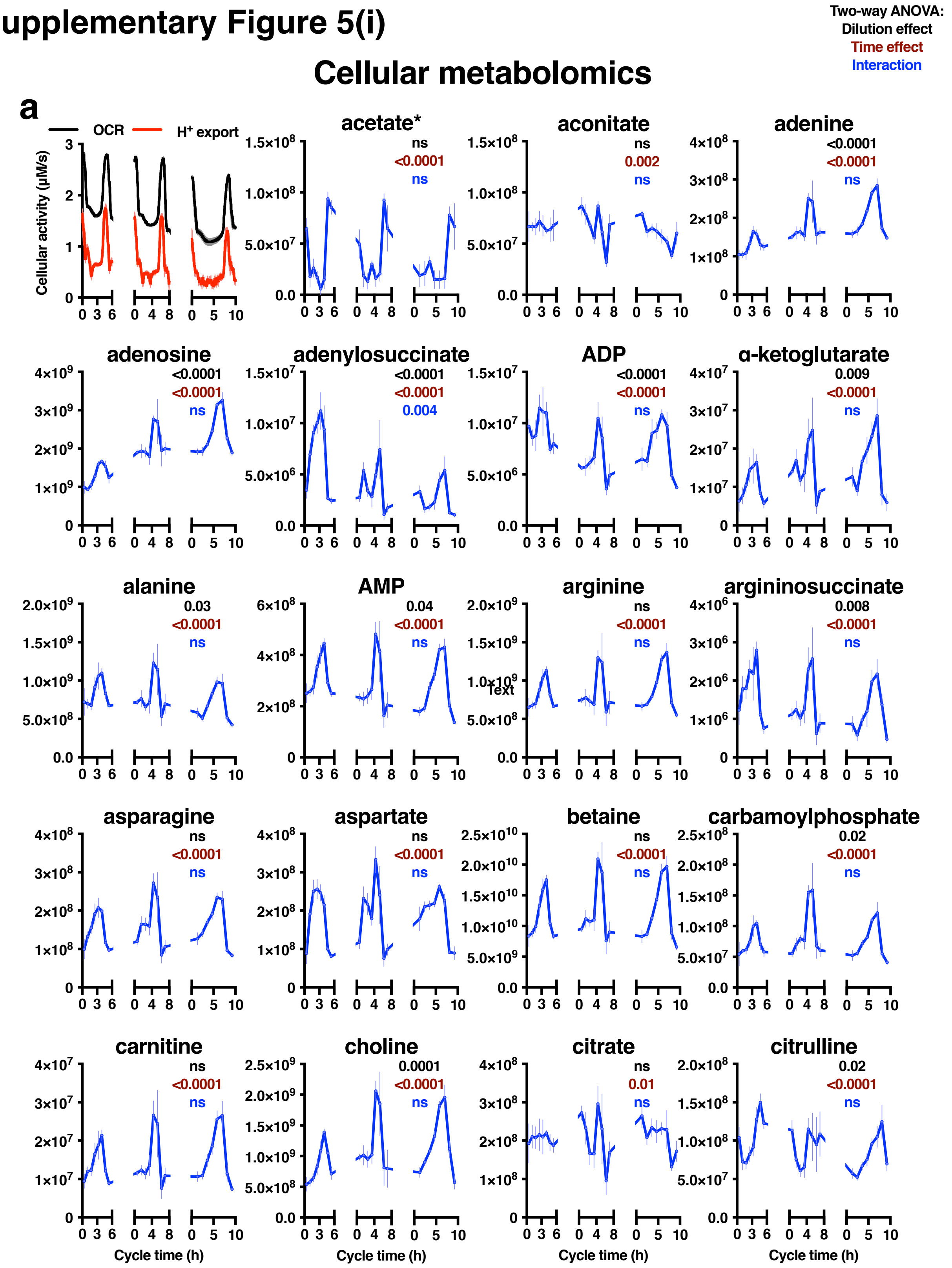

Supplementary Figure 5(ii)

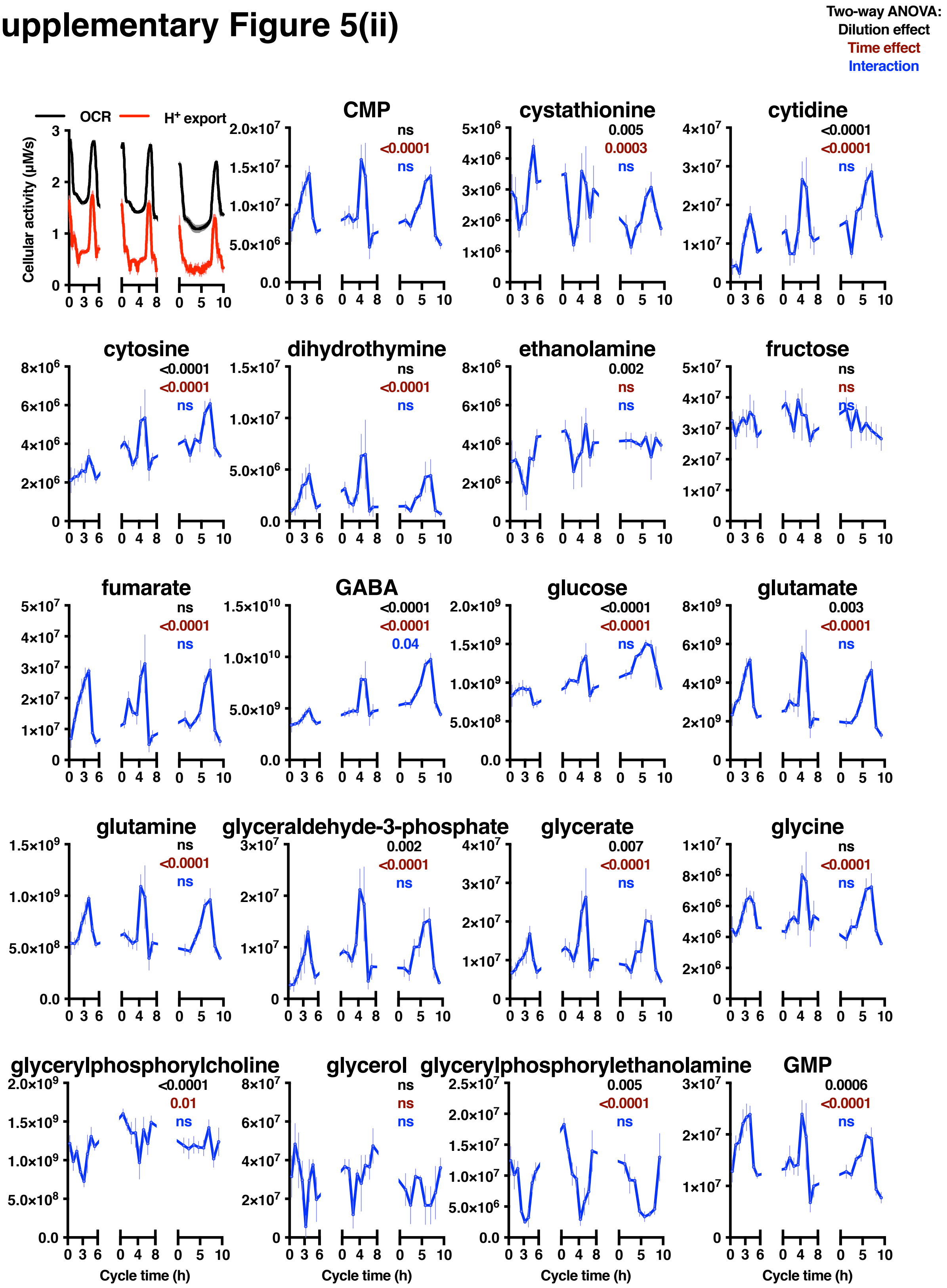

Supplementary Figure 5(iii)

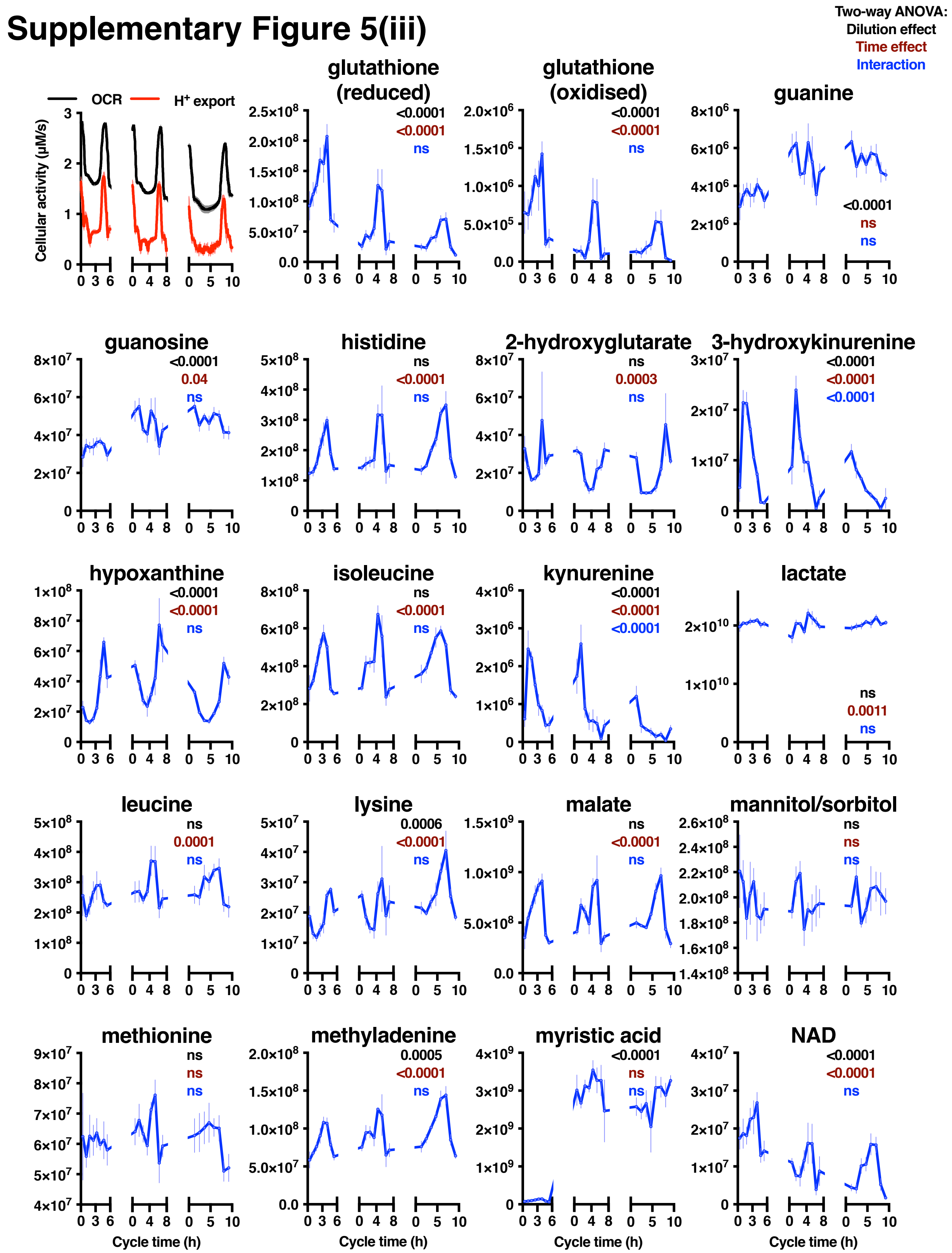

Supplementary Figure 5(iv)

Two-way ANOVA:
Dilution effect
Time effect
Interaction

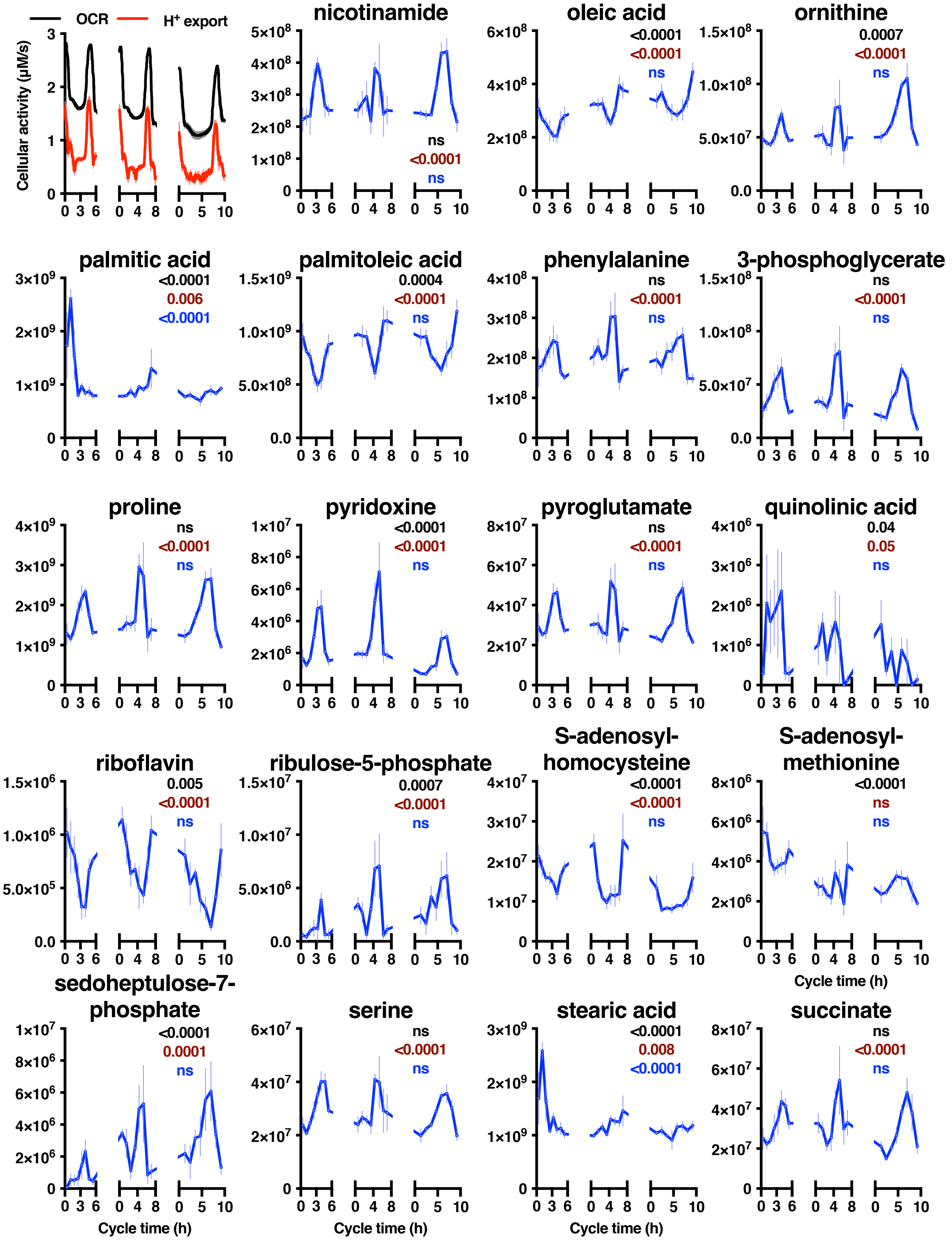

Supplementary Figure 5(v)

Two-way ANOVA:
Dilution effect
Time effect
Interaction

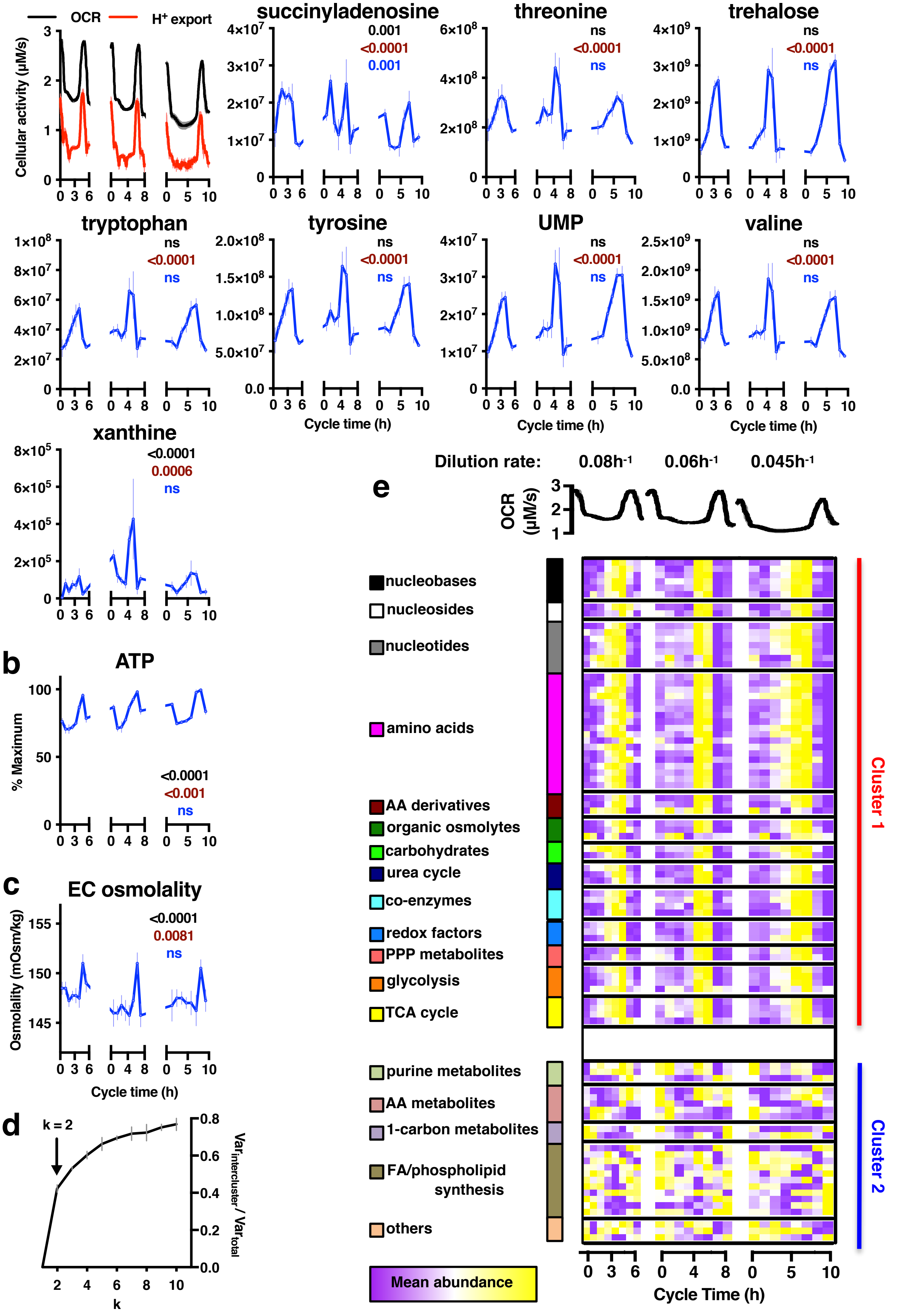

Supplementary Figure 5, Relating to Figure 2. Most cellular metabolites detected vary consistently over the YRO.

a, The abundance of most detected metabolites significantly and consistently varies across the YRO (mean±SEM, n=4 biological replicates, 2-way ANOVA reported). 82/89 of the metabolites identified in each replicate at every time point showed a time effect by two-way ANOVA with $p<0.05$, we describe these as consistently rhythmic N.B. unlike the other metabolites, acetate was not validated against external standards but its profile is entirely consistent with previous reports[3]. **b**, Cellular ATP content varies over the YRO, measured enzymatically as previously[46] (mean±SEM, n=4 biological replicates, 2-way ANOVA reported). **c**, The osmolality of the extracellular media (EC osmolality) transiently increases during HOC (mean±SEM, n=4 biological replicates, 2-way ANOVA reported). **d**, As with consistently rhythmic protein profiles (Supplementary Figure 2b), unbiased k means cluster analysis of consistently rhythmic metabolites suggests that two clusters of temporal profile are appropriate, as revealed by the 'break point' in a plot of inter-cluster variation versus cluster number. **e**, Heat map for consistently rhythmic metabolites associated with Cluster 1 or 2, normalized to the average minimum and maximum detected abundance of each metabolite for each YRO cycle. Cluster 2 suggests an association of phospholipid synthesis with LOC, whereas profiles of metabolites in cluster 1 are similar to K^+, peaking around the LOC=>HOC transition and falling as OCR increases. This includes amino acids, organic osmolytes and storage carbohydrates.

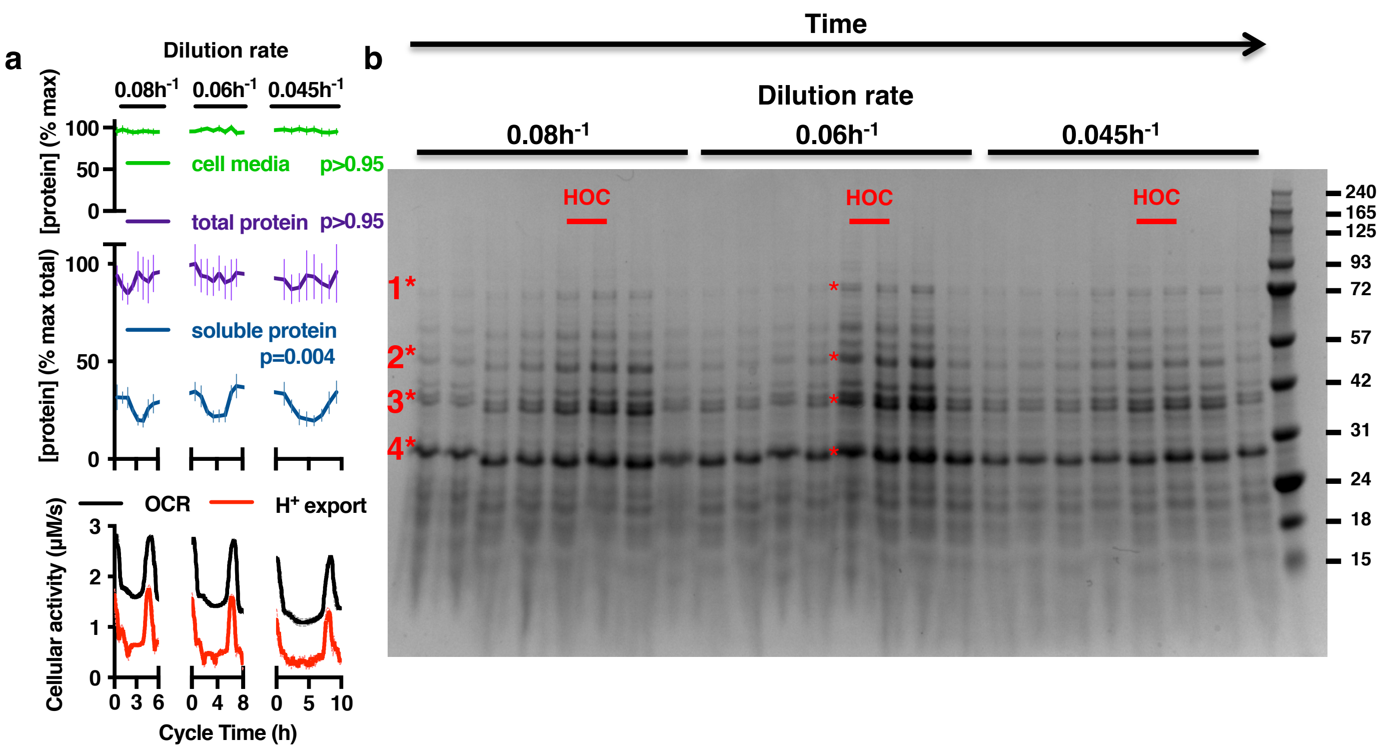

c

Band	Enriched for GO term	Proteins with GO annotation
1	Protein-binding involved in protein folding, p = 2.06 x 10^{-13}	Ssa2, Ssa3, Ssc1, Kar2, Ssa1, Ssa4
2	Catalytic activity, p = 4.56 x 10^{-11}	Atp2, Erg6, Arc1, Met17, Pdx1, Err3, Eno2, Arg1, Idp3, Hsp60, Eno1, Cit1, Pot1, Mri1, Pmi40, Sam1, Pda1, Gdh1, Kgd2, Pgk1, Tef2, Ald4, Tuf1, Idp1, Ddi1, Lys9, Cor1, Sah1, Pdc1, Hat2, Sam2, Rpt6, Oye3, Cdc19, Cpr6
3	Oxidoreductase activity, p = 3.63 x 10^{-14}	Mtd1, Mdh1, Adh2, Zta1, Tdh2, Gre3, Hom6, Ypr1, Mrp1, Tdh3, Gcy1, Tdh1, Ara1, Aim45, Ydl124w, Lia1, Ypr127w, Adh1
4	Proteasome/endopeptidase activity, p = 2.04 x 10^{-6}	Pre9, Pre8, Scl1, Pre7, Pup2
4*	40S ribosomal subunit, ns (p = 0.06)	Rps3, Rps5, Rps0b, Rps8b

Supplementary Figure 6, Relating to Figure 2. Release of protein from cytosolic storage/stress granules, or BMCs, varies consistently over the YRO. a, The amount of protein resolubilized in reducing 8 M urea buffer following hot ethanol precipitation (soluble protein), from the equal numbers of cells, changes by more than 2-fold across the YRO, while the concentration of total cellular protein, or protein in the media, does not change significantly (mean±SEM, n=4 biological replicates, 2-way ANOVA time effect p-value reported). Precipitation in hot ethanol favours intramolecular disulphide formation for hydrated 'soluble proteins' in the cytosolic compartment, compared with an increased relative likelihood of intermolecular disulphide formation for proteins in BMCs. Upon reconstitution in reducing 8 M urea buffer at 37°C, most BMC disulphides are less solvent-accessible to the reductant (10 mM TCEP), and more likely to remain within insoluble aggregates that are removed by centrifugation, whereas precipitated proteins with accessible disulphides that were not within proteinacious non-membrane bound are more likely to go into solution and therefore remain in the supernatant upon centrifugation[5]. **b**, Representative coomassie-stained gel from n=4 experiments showing soluble protein extracted from equal numbers of cells harvested across YRO cycles at three dilution rates. **c**, Mass-spectrometry of proteins with highest variation across the YRO (*labelled 1 to 4) reveals enrichment for gene ontology terms associated with protein folding and degradation, as well as catalytic and oxido-reductase activity. From the proteins that were identified in each band, the most enriched GO term (function) is reported, with corrected p-value and identified proteins. 4* Ribosomal proteins were identified in band 4, however, they are not significantly enriched, due to the large number of ribosome-associated proteins in cells at all times.

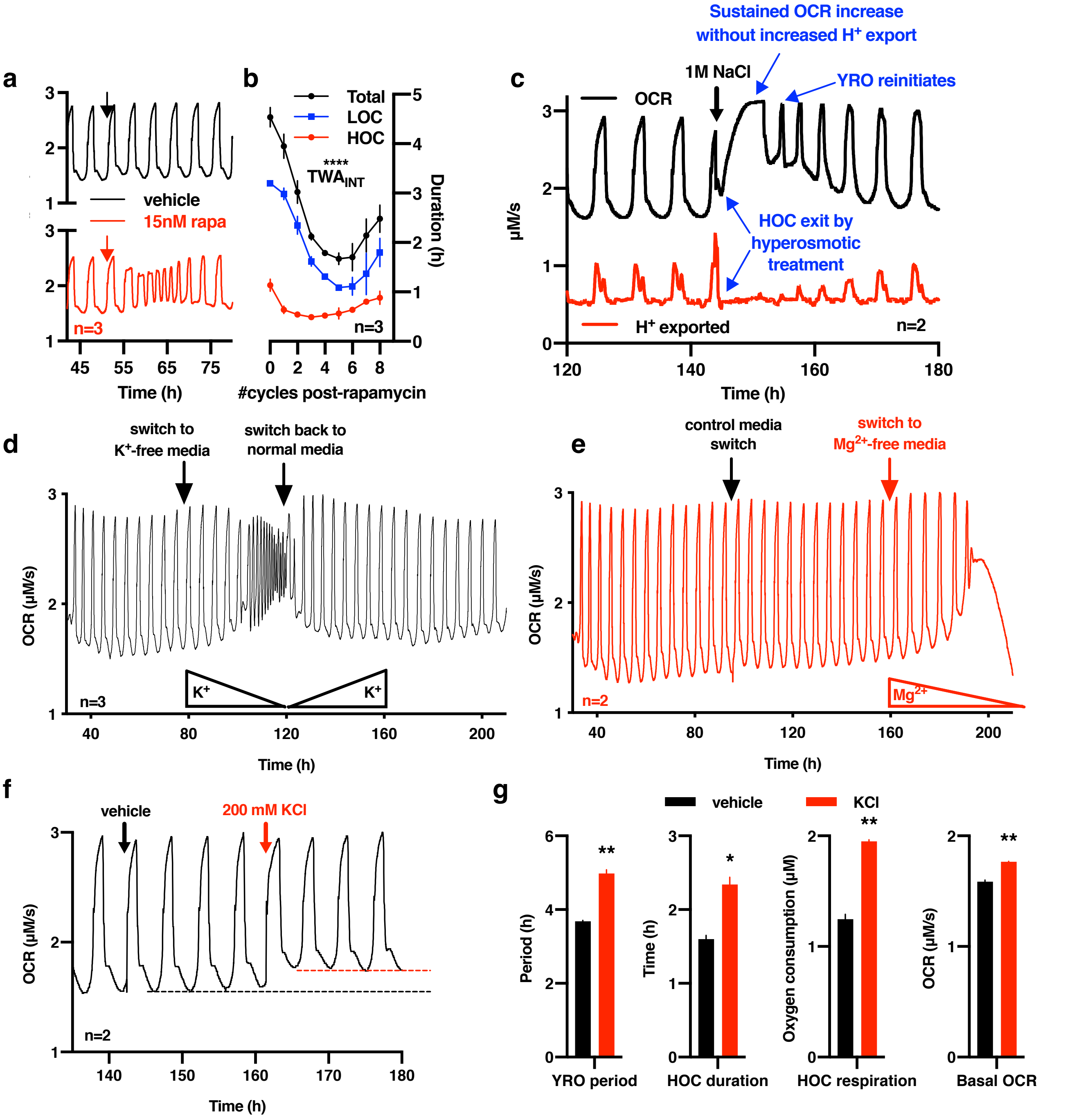

Supplementary Figure 7, Relating to Figures 3 and 4. Further experimental perturbation of the YRO. **a,b**, Inhibition of TORC1 activity during HOC by addition of rapamycin (rapa) transiently decreases the period and amplitude of oscillation. YROs recover as the drug is diluted out of the reactor vessel. The duration of LOC is affected more than HOC, likely because the resource required to support translation is not exhausted during HOC and is therefore replenished faster. Critically, the first LOC after rapamycin addition is not affected, whereas the next HOC is, consistent with differential regulation of TORC1 activity (n=3 independent experiments, TWA_{INT}, F (16, 48) = 13.58, p<0.0001). **c**, Osmotic stress promotes premature exit from HOC, likely due to increased macromolecular crowding, which inhibits TORC1 and opposes the liberation of proteins from BMCs. **d,e**, The effect of potassium depletion is ion-specific and is not due to loss of viability, as a return to standard inflow media (containing 14mM K^+) rapidly restores normal oscillations (n=3 independent experiments). **f, g**, The addition of KCl to cells undergoing HOC increases the duration of HOC, OCR and basal respiration rate, this is the opposite of the response to potassium depletion (n=2 independent experiments, t-tests from two oscillations per condition).

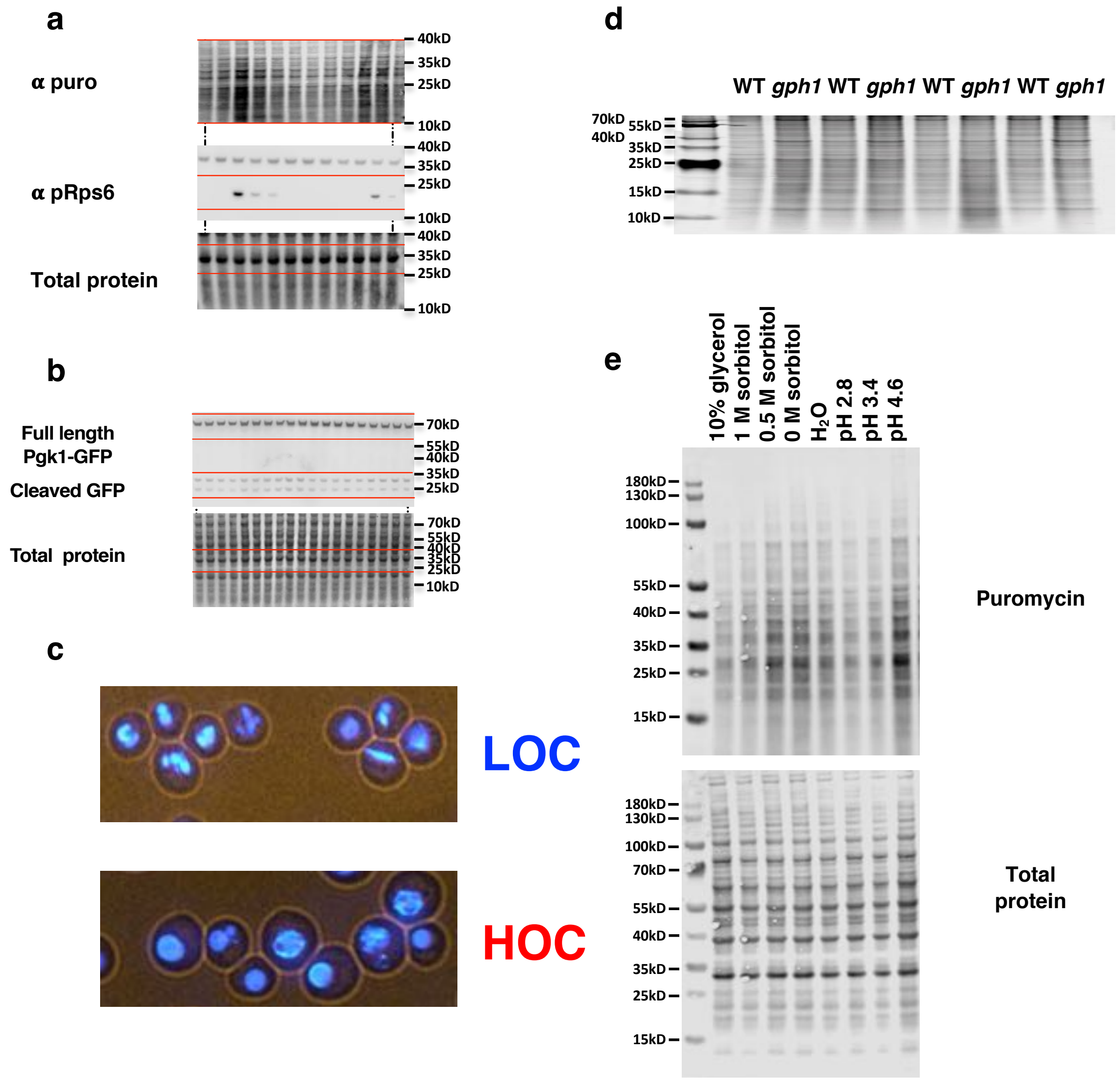

Supplementary Figure 8, Relating to Figures 3 and 5. Relationships between vacuole morphology and protein homeostasis with the YRO, extracellular osmolality and pH.

a, b Full immunoblots shown in Figure 3b and c. Red lines indicate the regions shown. **c**, Cells sampled from the bioreactor during LOC and HOC have vacuoles of different shapes (vacuoles stained with CMAC, representative of images quantified in Figure 3d). **d**, Strains deficient in the breakdown of glycogen (*gph1*) harbour more aggregated protein than wild type strains. Silver stained gel, quantified in Figure 5d, showing data from 4 biological replicates. **e**, Cells sampled from the bioreactor in mid-HOC incorporate less puromycin when diluted into media containing 10% glycerol or 1 M sorbitol, while cells sampled during early HOC incorporate less puromycin when diluted into media of pH 2.8 and more puromycin when diluted into media of pH 4.8. Data quantified in Fig. 5b.

A detailed, testable and experimentally-derived YRO model.
During exponential growth glucose is not limiting. Protein synthesis and growth (biomass accumulation, DNA synthesis, fatty acid synthesis) do not compete for bioenergetic resources.

A. Early LOC.

- Storage carbohydrates and amino acids are depleted, glucose supply is low (Fig.2a, [6-14]).
- Low glucose inhibits Pma1, the ATP-dependent plasma membrane H+-pump in yeast, resulting in a low rate of H+-export [15-22].
- Cytosolic pH drops (Fig.2c, Supplementary Fig.4a), coordinately regulating the activity of many metabolic and signalling pathways [23] e.g. inhibition of glycolysis [24].
- Low glucose availability and low cytosolic pH leads to a reduction in glycolytic flux and O_2 consumption, with decreased rates of ATP production [10,25] and a concomitant fall in energy charge (Fig.2a,c, Supplementary Fig.5).
- TORC1 is inactivated (Fig.3b) due to: (1) low energy charge *via* SNF1/AMPK (Fig.2a, Supplementary Fig.5, [26]); (2) amino acid depletion (Fig.2a, Supplementary Fig.5) via Gcn2 [27]; (3) inhibition of the activating GTPase Gtr1/2 due to low pH [28,29].
- TORC1 inactivation results in decreased protein synthesis (Fig.3b, [27]) and stimulates autophagy (Fig.3c, [30]).

B&C. Mid-Late LOC

- Pma1 activity is further limited by reduced ATP availability 20,21,23.
- Cytosol stabilizes at ~pH6.3, initiating a cellular response to starvation stress which facilitates macromolecular assembly of cytosolic enzymes and increased formation of biomolecular condensates such as stress granules and p-bodies (Fig.2c, Fig.4a, [22,31-41]). This resembles the quiescent state in which stress-resistance is enhanced and the cytoplasm is viscous (Fig.5a [7,42-44]).*
- TORC1 inactive (Fig.3b), reduced protein synthesis (Fig.3b), increased autophagy (Fig.3c), low oxygen consumption (Fig.2a) and energy charge (Fig.2a, Supplementary Fig.5).
- Sequestration of cytosolic metabolic enzymes such as Cdc19 [39] (Supplementary Fig.6b,c), low pH and reduced glycolytic flux [24] direct incoming glucose toward production of storage carbohydrate (glycogen and trehalose, Fig.2a, [7,10-13,38,45]), and generate biosynthetic intermediates for cell growth via the pentose phosphate pathway, fatty acid and DNA synthesis (Supplementary Fig1, Supplementary Fig5, [46]). The ~1000-fold H+ gradient (pH 3.4 extracellularly) across the plasma membrane is used in secondary active transport to accumulate nutrients and osmolytes (Fig.2a,b, [20,47]).
- Import of K+ and other osmolytes (Fig.2a, Supplementary Fig.4c) counter-balances the reduced contribution that sequestered cytosolic macromolecules make to cellular osmotic potential [47,48]. The cytosol is predicted to be 'glass-like' [42,44,49,50].
- Autophagy and amino acid symporters act to replenish vacuolar amino acid stores [51,52].

D. Entry to HOC

- Replete carbohydrate stores (Fig.2a, 4b) stimulate glycolysis, respiration and ATP production [53,54].
- Increased ATP availability and higher glycolytic flux increase Pma1 activity [20,21,23].
- Cytosolic pH begins to increase (Fig.2a,c).
- Energy charge increases due to increased ATP production relative to consumption and associated fall in AMP/ADP (Fig.2a, Supplementary Fig.5).
- Increased energy charge relieves SNF1/AMPK-mediated inhibition of TORC1 [26] and stimulates glycogenolysis [53].
- Replete amino acid stores relieve Gcn2-mediated inhibition of TORC1 [27].
- Condensate disassembly is initially attenuated by high cytosolic osmolyte concentration (K+, choline, betaine) and low pH (Fig.2a,c; Supplementary Figs.4,5).

E. Early HOC

- Elevated cytosolic pH and increasing energy charge trigger the release of sequestered ribosomes, proteasomes, chaperones and metabolic enzymes such as Cdc19 from macromolecular assemblies and condensates [31-40,55] – a feed-forward switch that further stimulates glycolysis (Supplementary Fig.5) and glycogen/trehalose breakdown (Fig.4b, Supplementary Fig.5, [56]). This increases ATP production and the rate of H+-export (Fig.2a,c, [18,21,57]. Cytosolic pH reaches pH7 (Fig.2c).
- Increased cytosolic pH activates TORC1 [28,29].
- TORC1 activation stimulates increased translational initiation and represses autophagy [27,30,58].
- To maintain osmotic homeostasis, osmolytes are exported down their concentration gradients. This buffers the increase in osmotic potential resulting from the increase in cytosolic macromolecules, and the cytosol becomes more fluid [42,44,47-50].

F. Late HOC

- Glycogen and trehalose breakdown sustain high glycolytic flux and respiration (Fig.2a, 4b, Supplementary Fig.5).
- Increased mitochondrial ATP production rate is stimulated by, and sustains, the high rate of ATP turnover required for efficient protein synthesis [59,60]. The proportion of ribosomes available for translation increases (Supplementary Fig.6, [44]).
- Continued export of osmolytes counter-balances the increase in osmotic potential due to newly synthesized macromolecules [44,47,48].
- Increased protein synthesis consumes stored amino acids (Fig.2a, Supplementary Fig.6)
- DNA replication and fatty acid synthesis does not occur (Supplementary Fig.1f,g) as cellular glucose is used to sustain translational bursting, which now consumes up to 75% of cellular energy [59,61,62].

G. HOC Exit

- The decrease in protein synthesis leads to decreased ATP turnover and oxygen consumption falls (Fig.2a, 3b, Supplementary Fig.5b).
- This could be due to insufficient: stored osmolytes to buffer further protein synthesis (equivalent to osmotic stress, Fig.2a, Supplementary Fig.5 [63]), stored amino acids to sustain further protein synthesis [27]; stored carbohydrates or oxygen to meet the requirements of protein synthesis and H+-export (via TORC1, [26,28,29]).
- This predicts that premature HOC exit will occur on acute osmotic stress (Supplementary Fig.7c), inhibition of protein synthesis or inhibition of TORC1 activity (Fig.3e, Supplementary Figure 7a,b) and that perturbation of osmotic buffering capacity, the transmembrane H+-gradient, or Pma1 activity will alter the period of oscillation (Fig. 4c-h, Supplementary Figure 7c-g).

*The mechanisms that facilitate widespread macromolecular assembly formation and condensation in low glucose are incompletely understood, but have been widely observed, and are likely to involve increased association of proteins with RNA, and other proteins, through electrostatic charge-charge interactions made favourable by histidine protonation, lysine/arginine modification and changes in protein phosphorylation, as well as changes in ionic strength and the chemical potential of water [36,64-71].

Supplementary Table 1, Relating to Figure 5e

Supplementary References

1 Newman, J. R. et al. Single-cell proteomic analysis of S. cerevisiae reveals the architecture of biological noise. Nature 441, 840-846, (2006).

2 Klevecz, R. R., Bolen, J., Forrest, G. & Murray, D. B. A genomewide oscillation in transcription gates DNA replication and cell cycle. P.N.A.S (USA) 101, 1200-1205 (2004).

3 Tu, B. P., Kudlicki, A., Rowicka, M. & McKnight, S. L. Logic of the yeast metabolic cycle: temporal compartmentalization of cellular processes. Science 310, 1152-1158 (2005).

4 Wang, G. Z. et al. Cycling Transcriptional Networks Optimize Energy Utilization on a Genome Scale. Cell Reports 13, 1868-1880, (2015).

5 Damodaran, S. in Food Proteins and Their Applications (Imprint Routledge, New York, 2017).

6 Sillje, H. H. et al. Function of trehalose and glycogen in cell cycle progression and cell viability in Saccharomyces cerevisiae. Journal of bacteriology 181, 396-400 (1999).

7 Wang, J. et al. Cellular stress responses oscillate in synchronization with the ultradian oscillation of energy metabolism in the yeast Saccharomyces cerevisiae. FEMS Microbiol. Lett. 189, 9-13 (2000).

8 Muller, D., Exler, S., Aguilera-Vazquez, L., Guerrero-Martin, E. & Reuss, M. Cyclic AMP mediates the cell cycle dynamics of energy metabolism in Saccharomyces cerevisiae. Yeast 20, 351-367, (2003).

9 Futcher, B. Metabolic cycle, cell cycle, and the finishing kick to Start. Genome biology 7, 107, (2006).

10 Xu, Z. & Tsurugi, K. A potential mechanism of energy-metabolism oscillation in an aerobic chemostat culture of the yeast Saccharomyces cerevisiae. The FEBS journal 273, 1696-1709, (2006).

11 Tu, B. P. et al. Cyclic changes in metabolic state during the life of a yeast cell. P.N.A.S (USA) 104, 16886-16891 (2007).

12 Xu, Z. & Tsurugi, K. Destabilization of energy-metabolism oscillation in the absence of trehalose synthesis in the chemostat culture of yeast. Archives of Biochemistry and Biophysics 464, 350-358, (2007).

13 Ouyang, Y., Xu, Q., Mitsui, K., Motizuki, M. & Xu, Z. PSK2 coordinates glucose metabolism and utilization to maintain ultradian clock-coupled respiratory oscillation in Saccharomyces cerevisiae yeast. Archives of Biochemistry and Biophysics 509, 52-58, (2011).

14 Chin, S. L., Marcus, I. M., Klevecz, R. R. & Li, C. M. Dynamics of oscillatory phenotypes in Saccharomyces cerevisiae reveal a network of genome-wide transcriptional oscillators. The FEBS Journal 279, 1119-1130, (2012).

15 Serrano, R. In vivo glucose activation of the yeast plasma membrane ATPase. FEBS letters 156, 11-14, (1983).

16 Serrano, R., Kielland-Brandt, M. C. & Fink, G. R. Yeast plasma membrane ATPase is essential for growth and has homology with (Na+ + K+), K+- and Ca2+-ATPases. Nature 319, 689-693, (1986).

17 Eraso, P. & Gancedo, C. Activation of yeast plasma membrane ATPase by acid pH during growth. FEBS letters 224, 187-192, (1987).

18 Souza, M. A. A., Tropia, M. J. & Brandao, R. L. New aspects of the glucose activation of the H(+)-ATPase in the yeast Saccharomyces cerevisiae. Microbiology 147, 2849-2855, (2001).

19 Permyakov, S., Suzina, N. & Valiakhmetov, A. Activation of H+-ATPase of the plasma membrane of Saccharomyces cerevisiae by glucose: the role of sphingolipid and lateral enzyme mobility. PLoS One 7, e30966, (2012).

20 Volkov, V. Quantitative description of ion transport via plasma membrane of yeast and small cells. Front Plant Sci 6, 425, (2015).

21 Kane, P. M. Proton Transport and pH Control in Fungi. Advances in experimental medicine and biology 892, 33-68, (2016).

22 Peters, L. Z., Hazan, R., Breker, M., Schuldiner, M. & Ben-Aroya, S. Formation and dissociation of proteasome storage granules are regulated by cytosolic pH. J Cell Biol 201, 663-671, (2013).

23 Orij, R., Brul, S. & Smits, G. J. Intracellular pH is a tightly controlled signal in yeast. Biochim Biophys Acta 1810, 933-944, (2011).

24 Krebs, H. A., Wiggins, D., Stubbs, M., Sols, A. & Bedoya, F. Studies on the mechanism of the antifungal action of benzoate. The Biochemical journal 214, 657-663, (1983).

25 Satroutdinov, A. D., Kuriyama, H. & Kobayashi, H. Oscillatory metabolism of Saccharomyces cerevisiae in continuous culture. FEMS Microbiol Lett 98, 261-268 (1992).

26 DeMille, D. et al. PAS kinase is activated by direct SNF1-dependent phosphorylation and mediates inhibition of TORC1 through the phosphorylation and activation of Pbp1. Mol Biol Cell 26, 569-582, (2015).

27 Yuan, W. et al. General Control Nonderepressible 2 (GCN2) Kinase Inhibits Target of Rapamycin Complex 1 in Response to Amino Acid Starvation in Saccharomyces cerevisiae. J Biol Chem 292, 2660-2669, (2017).

28 Dechant, R., Saad, S., Ibanez, A. J. & Peter, M. Cytosolic pH regulates cell growth through distinct GTPases, Arf1 and Gtr1, to promote Ras/PKA and TORC1 activity. Mol Cell 55, 409-421, (2014).

29 Saliba, E. et al. The yeast H(+)-ATPase Pma1 promotes Rag/Gtr-dependent TORC1 activation in response to H(+)-coupled nutrient uptake. eLife 7, doi:10.7554/eLife.31981 (2018).

30 Hu, Z. et al. Multilayered Control of Protein Turnover by TORC1 and Atg1. Cell reports 28, 3486-3496 e3486, (2019).

31 Buchan, J. R. & Parker, R. Eukaryotic stress granules: the ins and outs of translation. Mol Cell 36, 932-941, (2009).

32 Grousl, T. et al. Robust heat shock induces eIF2alpha-phosphorylation-independent assembly of stress granules containing eIF3 and 40S ribosomal subunits in budding yeast, Saccharomyces cerevisiae. J Cell Sci 122, 2078-2088, (2009).

33 Iwaki, A. & Izawa, S. Acidic stress induces the formation of P-bodies, but not stress granules, with mild attenuation of bulk translation in Saccharomyces cerevisiae. The Biochemical Journal 446, 225-233, (2012).

34 Zid, B. M. & O'Shea, E. K. Promoter sequences direct cytoplasmic localization and translation of mRNAs during starvation in yeast. Nature 514, 117-121, (2014).

35 Munder, M. C. et al. A pH-driven transition of the cytoplasm from a fluid- to a solid-like state promotes entry into dormancy. eLife 5, doi:10.7554/eLife.09347 (2016).

36 Banani, S. F., Lee, H. O., Hyman, A. A. & Rosen, M. K. Biomolecular condensates: organizers of cellular biochemistry. Nature reviews. Molecular cell biology 18, 285-298, (2017).

37 Jin, M. et al. Glycolytic Enzymes Coalesce in G Bodies under Hypoxic Stress. Cell Reports 20, 895-908, (2017).

38 Saad, S. et al. Reversible protein aggregation is a protective mechanism to ensure cell cycle restart after stress. Nature Cell Biology 19, 1202-1213, (2017).

39 Prouteau, M. & Loewith, R. Regulation of Cellular Metabolism through Phase Separation of Enzymes. Biomolecules 8, doi:10.3390/biom8040160 (2018).

40 van Leeuwen, W. & Rabouille, C. Cellular stress leads to the formation of membraneless stress assemblies in eukaryotic cells. Traffic 20, 623-638, (2019).

41 Marshall, R. S. & Vierstra, R. D. Proteasome storage granules protect proteasomes from autophagic degradation upon carbon starvation. eLife 7, doi:10.7554/eLife.34532 (2018).

42 Parry, B. R. et al. The bacterial cytoplasm has glass-like properties and is fluidized by metabolic activity. Cell 156, 183-194, (2014).

43 Lavut, A. & Raveh, D. Sequestration of highly expressed mRNAs in cytoplasmic granules, P-bodies, and stress granules enhances cell viability. PLoS Genet 8, e1002527, (2012).

44 Delarue, M. et al. mTORC1 Controls Phase Separation and the Biophysical Properties of the Cytoplasm by Tuning Crowding. Cell 174, 338-349 e320, (2018).

45 Shi, L., Sutter, B. M., Ye, X. & Tu, B. P. Trehalose is a key determinant of the quiescent metabolic state that fuels cell cycle progression upon return to growth. Mol Biol Cell 21, 1982-1990, (2010).

46 Casanovas, A. et al. Quantitative analysis of proteome and lipidome dynamics reveals functional regulation of global lipid metabolism. Chem Biol 22, 412-425, (2015).

47 Arino, J., Ramos, J. & Sychrova, H. Monovalent cation transporters at the plasma membrane in yeasts. Yeast 36, 177-193, (2019).

48 Saxena, A. & Sitaraman, R. Osmoregulation in Saccharomyces cerevisiae via mechanisms other than the high-osmolarity glycerol pathway. Microbiology 162, 1511-1526, (2016).

49 Joyner, R. P. et al. A glucose-starvation response regulates the diffusion of macromolecules. eLife 5, doi:10.7554/eLife.09376 (2016).

50 Heimlicher, M. B. et al. Reversible solidification of fission yeast cytoplasm after prolonged nutrient starvation. J Cell Sci 132, (2019).

51 Ohsumi, Y. Historical landmarks of autophagy research. Cell Res 24, 9-23, (2014).

52 Kawano-Kawada, M., Kakinuma, Y. & Sekito, T. Transport of Amino Acids across the Vacuolar Membrane of Yeast: Its Mechanism and Physiological Role. Biological & Pharmaceutical Bulletin 41, 1496-1501, (2018).

53 Wilson, W. A. et al. Regulation of glycogen metabolism in yeast and bacteria. FEMS microbiology reviews 34, 952-985, (2010).

54 Nilsson, A. & Nielsen, J. Metabolic Trade-offs in Yeast are Caused by F1F0-ATP synthase. Scientific reports 6, 22264, (2016).

55 Narayanaswamy, R. et al. Widespread reorganization of metabolic enzymes into reversible assemblies upon nutrient starvation. Proc Natl Acad Sci U S A 106, 10147-10152, (2009).

56 Guillou, V., Plourde-Owobi, L., Parrou, J. L., Goma, G. & Francois, J. Role of reserve carbohydrates in the growth dynamics of Saccharomyces cerevisiae. FEMS Yeast Res 4, 773-787, (2004).

57 Hayek, S. R., Rane, H. S. & Parra, K. J. Reciprocal Regulation of V-ATPase and Glycolytic Pathway Elements in Health and Disease. Front Physiol 10, 127, (2019).

58 Parenteau, J. et al. Introns are mediators of cell response to starvation. Nature 565, 612-617, (2019).

59 Li, G. W., Burkhardt, D., Gross, C. & Weissman, J. S. Quantifying absolute protein synthesis rates reveals principles underlying allocation of cellular resources. Cell 157, 624-635, (2014).

60 Chen, X. et al. Cisplatin induces autophagy to enhance hepatitis B virus replication via activation of ROS/JNK and inhibition of the Akt/mTOR pathway. Free Radical Biology & Medicine 131, 225-236, (2019).

61 Dacheux, E., Firczuk, H. & McCarthy, J. E. Rate control in yeast protein synthesis at the population and single-cell levels. Biochemical Society Transactions 43, 1266-1270, (2015).

62 Stein, K. C. & Frydman, J. The stop-and-go traffic regulating protein biogenesis: How translation kinetics controls proteostasis. J Biol Chem 294, 2076-2084, (2019).

63 Uesono, Y. & Toh, E. A. Transient inhibition of translation initiation by osmotic stress. J Biol Chem 277, 13848-13855, (2002).

64 Riback, J. A. et al. Stress-Triggered Phase Separation Is an Adaptive, Evolutionarily Tuned Response. Cell 168, 1028-1040 e1019, (2017).

65 Monahan, Z. et al. Phosphorylation of the FUS low-complexity domain disrupts phase separation, aggregation, and toxicity. The EMBO Journal 36, 2951-2967, (2017).

66 Shively, C. A. et al. Large-Scale Analysis of Kinase Signaling in Yeast Pseudohyphal Development Identifies Regulation of Ribonucleoprotein Granules. PLoS Genet 11, e1005564, (2015).

67 Hofweber, M. et al. Phase Separation of FUS Is Suppressed by Its Nuclear Import Receptor and Arginine Methylation. Cell 173, 706-719 e713, (2018).

68 Saito, M. et al. Acetylation of intrinsically disordered regions regulates phase separation. Nature Chemical Biology 15, 51-61, (2019).

69 Owen, I. & Shewmaker, F. The Role of Post-Translational Modifications in the Phase Transitions of Intrinsically Disordered Proteins. Int J Mol Sci 20, (2019).

70 Falahati, H. & Haji-Akbari, A. Thermodynamically driven assemblies and liquid-liquid phase separations in biology. Soft Matter 15, 1135-1154, (2019).

71 Bah, A. & Forman-Kay, J. D. Modulation of Intrinsically Disordered Protein Function by Post-translational Modifications. J Biol Chem 291, 6696-6705, (2016).
