# Supplementary material for: Eukaryotic cell biology is temporally coordinated to support the energetic demands of protein homeostasis
Source: Nat Commun. 2020 Sep 17;11:4706. doi: 10.1038/s41467-020-18330-x (PMC7499178; doi:10.1038/s41467-020-18330-x)
Supplement: Supplementary file 2 — Peer Review File [file 41467_2020_18330_MOESM2_ESM.docx]

Peer Review Information

**Journal:** Nature Communications

**Manuscript title:** Dr Helen Causton

**Corresponding author name(s):** Eukaryotic cell biology is temporally coordinated to support the energetic demands of protein homeostasis

**Editorial notes:**

**Transferred manuscripts**

This manuscript has been previously reviewed at another journal that is not operating a transparent peer review scheme. This document only contains reviewer comments and rebuttal letters for versions considered at *Nature Communications*.

**Reviewer comments & decisions:**

**Reviewer comments, first version:**

REVIEWERS' COMMENTS:

Reviewer #1 (Remarks to the Author):

O’Neill et al provide an unparalleled characterization of yeast metabolic cycles that occur in batch cultures upon nutrient limiting conditions. The provided data establish a detailed understanding of the mechanisms underlying these oscillations and the authors test some of the derived hypothesis to further confirm the model. Thus, the manuscript represents a major contribution leading to better understanding of metabolic oscillations in yeast, which resemble metabolic oscillations in mammalian cells and thus will be of broad interest.

In particular, the authors have made considerable effort to further improve the manuscript and have fully addressed all my previous points. Thus, I fully support publication of the manuscript in its current form.


Reviewer #2 (Remarks to the Author):

The manuscript is significantly improved and is much easy to understand now. There are some minor changes to be done such as In Ext Fig1g the time intervals are not defined. Extended data figure 2 and 3, as well as fig 6 and 7 might be placed in an opposite order. Also the way the cells in the culture are synchronised might be better explain. Other than that the work is interesting, convincing and worthy to be published in Nature Communications.


Reviewer #3 (Remarks to the Author):

I reviewed this manuscript before and repeat here essentially what I had written previously:
The data presented by O’Neill et al. support the model that yeast respiratory oscillations are a cellular strategy to temporally coordinate metabolism under conditions of limiting resources in order to optimize protein biosynthesis. Thus, low oxygen consumption demarcates a phase of sequestration and storage of resources (carbohydrates and amino acids), which are then spend during the subsequent high oxygen consumption phase to support a burst of protein synthesis. The authors provide a comprehensive and impressive narrative describing convincingly the molecular processes governing YROs and driving the transitions between HOCs and LOCs. They suggest that during LOC, low ATP level limit the activity of Pma1, leading to a drop in cytosolic pH. Low pH, low ATP and depleted amino acid stores inhibit TORC1 and promote sequestration of glycolytic enzymes and biosynthetic machinery in biomolecular condensates, balanced by an increase in osmolytes. In this phase, available resources are used to preferentially synthesize lipids and nucleotides rather than proteins. Surplus resources are stored until the maximal storage capacity is reached, the time for this depending on available nutrients. When stores are full, additional resources are metabolized, which initiates a feed forward loop: Respiration is stimulated, ATP raises, Pma1 pumps protons, cytosolic pH increases, promoting the liberation of glycolytic enzymes from BMCs. TORC1 is activated (due to higher pH and indirectly by vacuolar amino acids), and supports a burst of translation. When resources are used up, mitochondrial ATP synthesis drops, Pma1 activity is reduced and cytosolic pH decreases. TORC1 is inactivated, proteins are sequestered in BMCs, and osmolytes are produced and/or taken up to maintain osmotic potential. This is a wonderful and convincing model, which is supported by many previously published data and by data presented by the authors. Thus, the authors show that mutants defective in glycogen synthesis and breakdown did not support YROs. A hsp30 mutant, displaying elevated Pma1 activity showed truncated respiratory cycles. Inhibition of protein synthesis by CHX affected HOC, suggesting that HOC is driven by the energetic demand of protein synthesis. Similarly, partial inhibition of TORC1 by rapamycin affected HOCs but not LOCs (since TORC is already inactive). Analysis of puromycin incorporation indicates bursts of protein synthesis during HOCs. Analysis of lysosomes indicates rhythmic changes in size, analysis of S6 indicates rhythmic TORC, and so on.
I had previously asked for a more detailed model and the authors have provided this in the present version. I want to point out specifically that the paper contains a lot of beautiful data and asking the authors for additional revisions at this point would not make the paper any better.

Michael Brunner

**Author rebuttal, first version:**

REVIEWERS' COMMENTS:

Reviewer #1 (Remarks to the Author):

O’Neill et al provide an unparalleled characterization of yeast metabolic cycles that occur in batch cultures upon nutrient limiting conditions. The provided data establish a detailed understanding of the mechanisms underlying these oscillations and the authors test some of the derived hypothesis to further confirm the model. Thus, the manuscript represents a major contribution leading to better understanding of metabolic oscillations in yeast, which resemble metabolic oscillations in mammalian cells and thus will be of broad interest.

In particular, the authors have made considerable effort to further improve the manuscript and have fully addressed all my previous points. Thus, I fully support publication of the manuscript in its current form.

Reviewer #2 (Remarks to the Author):

The manuscript is significantly improved and is much easy to understand now. There are some

minor changes to be done such as In

Ext Fig1g the time intervals are not defined.

Thank you for pointing this out. We have now added the missing labels.

Extended data figure 2 and 3, as well as fig 6 and 7 might be placed in an opposite order. We elected to keep the order of Supplementary Figures 2 and 3, however, we have moved the detailed model from Extended Figure 7 into the main text (Figure 5e), so that the paper concludes with a visual representation describing the proposed mechanism. References to additional literature supporting the model are now provided in Supplementary Table 1.

Also the way the cells in the culture are synchronised might be better explain.

Text has been added to the Methods to explain how the culture was synchronised. It now reads, ‘To initiate YROs bioreactor vessels were each inoculated with 100 OD (A600) units/L cells in stationary phase. The culture was allowed grow and return to stationary phase and maintained

without addition of new media for 6 hours. Oscillations were initiated by refeeding the culture at the dilution rates above and allowed to stabilize for 1-2 days before data collection or harvesting of samples’.

Other than that the work is interesting, convincing and worthy to be published in Nature Communications.

Reviewer #3 (Remarks to the Author):

I reviewed this manuscript before and repeat here essentially what I had written previously: The data presented by O’Neill et al. support the model that yeast respiratory oscillations are a cellular strategy to temporally coordinate metabolism under conditions of limiting resources in order to optimize protein biosynthesis. Thus, low oxygen consumption demarcates a phase of sequestration and storage of resources (carbohydrates and amino acids), which are then spend during the subsequent high oxygen consumption phase to support a burst of protein synthesis. The authors provide a comprehensive and impressive narrative describing convincingly the molecular processes governing YROs and driving the transitions between HOCs and LOCs. They suggest that during LOC, low ATP level limit the activity of Pma1, leading to a drop in cytosolic pH. Low pH, low ATP and depleted amino acid stores inhibit TORC1 and promote sequestration of glycolytic enzymes and biosynthetic machinery in biomolecular condensates, balanced by an increase in osmolytes. In this phase, available resources are

used to preferentially synthesize lipids and nucleotides rather than proteins. Surplus resources are stored until the maximal storage capacity is reached, the time for this depending on available nutrients. When stores are full, additional resources are metabolized, which initiates a feed forward loop: Respiration is stimulated, ATP raises, Pma1 pumps protons, cytosolic pH increases, promoting the liberation of glycolytic enzymes from BMCs. TORC1 is activated (due to higher pH and indirectly by vacuolar amino acids), and supports a burst of translation. When resources are used up, mitochondrial ATP synthesis drops, Pma1 activity is reduced and cytosolic pH decreases. TORC1 is inactivated, proteins are sequestered in BMCs, and osmolytes are produced and/or taken up to maintain osmotic potential. This is a wonderful and convincing model, which is supported by many previously published data and by data presented by the authors. Thus, the authors show that mutants defective in

glycogen synthesis and breakdown did not support YROs. A hsp30 mutant, displaying elevated Pma1 activity showed truncated respiratory cycles. Inhibition of protein synthesis by CHX affected HOC, suggesting that HOC is driven by the energetic demand of protein synthesis. Similarly, partial inhibition of TORC1 by rapamycin affected HOCs but not LOCs (since TORC is already inactive). Analysis of puromycin incorporation indicates bursts of protein synthesis during HOCs. Analysis of lysosomes indicates rhythmic changes in size, analysis of S6 indicates rhythmic TORC, and so on.

I had previously asked for a more detailed model and the authors have provided this in the present version. I want to point out specifically that the paper contains a lot of beautiful data and

asking the authors for additional revisions at this point would not make the paper any better.

Michael Brunner

Thank you!

**Reviewer comments, second version:**

**Author rebuttal, second version:**

**Reviewer comments, third version:**

**Author rebuttal, third version:**
